# Boron-catalysed transition-metal-free arylation and alkenylation of allylic alcohols with boronic acids†

**DOI:** 10.1039/d2ra07919d

**Published:** 2023-01-23

**Authors:** Sixian Lu, Xingyu Chen, Xiaoqiang Chang, Shuaichen Zhang, Dong Zhang, Yifan Zhao, Lan Yang, Yue Ma, Peng Sun

**Affiliations:** a Institute of Chinese Materia Medica and Artemisinin Research Center, Academy of Chinese Medical Sciences Beijing 100700 China yma@icmm.ac.cn psun@icmm.ac.cn; b School of Pharmacy, Chengdu University Chengdu Sichuan 610106 China

## Abstract

The development of efficient catalytic reactions with excellent atom and step economy employing sustainable catalysts is highly sought-after in chemical synthesis to reduce the negative effects on the environment. The most commonly-used strategy to construct allylic compounds relies on the transition-metal-catalysed nucleophilic substitution reaction of allylic alcohol derivatives. These syntheses exhibit good yield and selectivity, albeit at the expense of toxic and expensive catalysts and extra steps. In this paper, we report a transition-metal-free arylation and alkenylation reaction between unprotected allylic alcohols and boronic acids. The reactions were performed with B(C_6_F_5_)_3_ as the catalyst in toluene, and corresponding products were obtained in 23–92% yields. The reaction has mild conditions, scalability, excellent atom and step economy.

## Abstract 2

B(C_6_F_5_)_3_-catalyzed cross-coupling reaction between allylic alcohols and boronic acids with excellent regioselectivity, broad scope and mild conditions.

## Graphical abstract

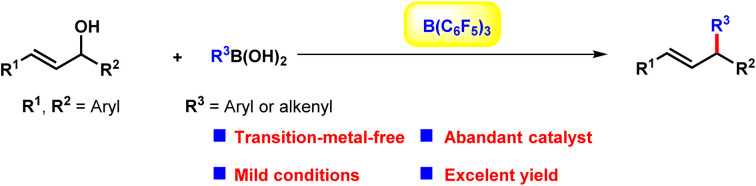

Allylic alkylation and alkenylation of allylic reagents such as allylic halides, carboxylates, carbonates, phosphates, and related compounds are some of the most important textbook reactions for carbon–carbon bond-forming, and have been widely applied in the syntheses of a broad scope of complex molecules.^1^ Among these dozens of building blocks, the direct transformation of allylic alcohols would be highly beneficial from the viewpoint of sustainable chemistry:^1*h*,2^ (a) the substrate could be used without the pre-activation of the hydroxyl group; (b) avoidance of other undesired by-products other than water. Regarding the other reaction partner, boronic acids^3^ are a promising choice compared with halides, grignard reagents and other feedstocks due to their stability, operational convenience, negligible toxicity, and broad functional group compatibility. A number of transition-metal-catalysed cross-coupling reactions between allylic alcohols and boronic acids have been established based on π-allyl metal complexes with outstanding efficacy and selectivity in a long-range.^4^ However, these cases bear the intrinsic limitations of the transition-metal-catalysts, which are widely regarded as expensive, hard to prepare, oxygen and moisture sensitive. As an alternative, several Brønsted and Lewis acids catalysed substitution reaction of allylic alcohols with different nucleophiles have been explored as the environmental benign approaches to obtain allylic compounds.^5^ In 2015, Gandon^6^ group reported a alkenylation of alcohols with vinylboronic acids employing air stable calcium(ii) complex as the catalyst. Despite of these progresses, the green and sustainable catalytic arylation protocol between alcohols and boronic acids remain underdeveloped. In the last decade, B(C_6_F_5_)_3_ has been realized to be capable of initiating a wide range of chemical transformations with remarkable performance.^7^ In particular, the cross-coupling reaction of alcohols to form C–C and C–P bond have been achieved with B(C_6_F_5_)_3_ by Xie^8^*et al.* very recently. Herein, as our longstanding interest in the development of environmental-friendly chemical transformations, we envisaged to explore the efficacy of B(C_6_F_5_)_3_ in the arylation and alkenylation reaction of allylic alcohols (Scheme 1).

**Scheme 1:**
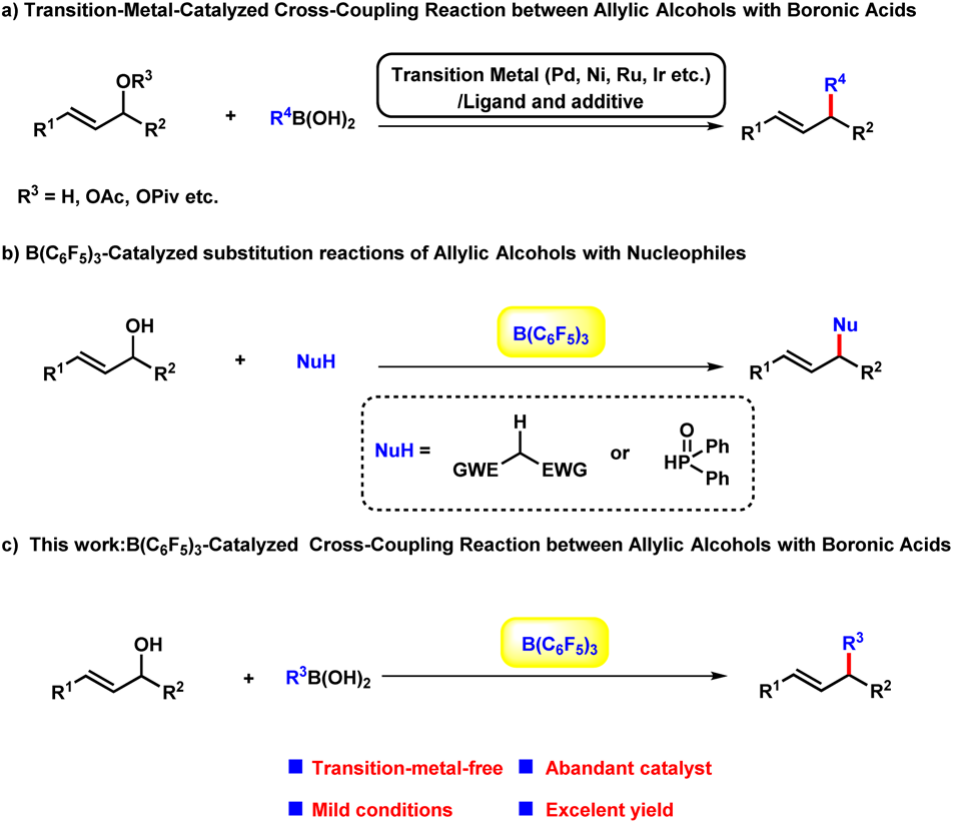
Catalytic cross-coupling reaction between boronic acids and allylic alcohol derivatives.

Initially, the (*E*)-1,3-diphenylprop-2-en-1-ol 1a and 4-methoxyphenylboronic acid 2a were chosen as the model substrates to optimize the reaction conditions. The reaction of 1a and 2a was carried out in the presence of B(C_6_F_5_)_3_ (20 mol%) at 80 °C in MeCN, affording compound 3a in 57% yield (Table 1, entry 1). Delighted by this initial result, we investigated the reaction conditions comprehensively. The screening of the solvent indicated that the yield could be promoted to 71% in nonpolar methylbenzene (Table 1, entry 2–7). Lewis acid catalysts such as Cu(OTf)_2_, Zn(OTf)_2_, and Sc(OTf)_2_ were evaluated to offer the desired product only in moderate yield (Table 1, entries 8–10). Moreover, other boron containing catalysts including B(OH)_3_ and BF_3_·OEt_2_ only offered diminished yields as low as 32% (Table 1, entries 11–12). In consideration of that the unstability of alcohols in presence of the strong Lewis acid catalyst, we increased 1a to 1.2 equivalent and more, up to 96% yield was obtained with 2.0 equivalent of 1a (Table 1, entries 13–15). The desired product could also be obtained in 93% yield with a reduced amount of B(C_6_F_5_)_3_ of 10% (Table 1, entries 16–17). Then, we conducted the reaction at lower temperature, and the results indicated that the reaction performed smoothly at ambient temperature giving 91% yield (Table 1, entries 18–19).

**Table 1:** The optimization of the conditions^a^

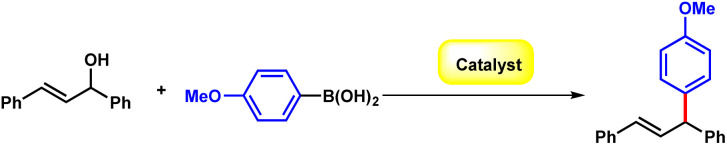				
Entry	Catalyst	Solvent	Temperature (°C)	Yield^b^
1	B(C_6_F_5_)_3_	CH_3_CN	80	57%
2	B(C_6_F_5_)_3_	DMF	80	38%
3	B(C_6_F_5_)_3_	DMSO	80	25%
4	B(C_6_F_5_)_3_	THF	80	18%
5	B(C_6_F_5_)_3_	DCE	80	62%
6	B(C_6_F_5_)_3_	EA	80	12%
7	B(C_6_F_5_)_3_	Toluene	80	71%
8	Cu(OTf)_2_	Toluene	80	55%
9	Zn(OTf)_2_	Toluene	80	52%
10	Sc(OTf)_2_	Toluene	80	63%
11	B(OH)_3_	Toluene	80	36%
12	BF_3_·OEt_2_	Toluene	80	32%
13^c^	B(C_6_F_5_)_3_	Toluene	80	88%
14^d^	B(C_6_F_5_)_3_	Toluene	80	91%
15^e^	B(C_6_F_5_)_3_	Toluene	80	96%
16^f^	B(C_6_F_5_)_3_	Toluene	80	93%
17^g^	B(C_6_F_5_)_3_	Toluene	80	85%
18	B(C_6_F_5_)_3_	Toluene	50	90%
19^h^	B(C_6_F_5_)_3_	Toluene	25	91%

a Reaction conditions: 1a (0.1 mmol), 2a (0.2 mmol), catalyst (20 mol%), solvent (2 mL), 6 hours, under air atmosphere.

b Isolated yield.

c 1.2 equiv. of 1a was added.

d 1.5 equiv. of 1a was added.

e 2.0 equiv. of 1a was added.

f 10 mol% of B(C_6_F_5_)_3_ was used.

g 5 mol% of B(C_6_F_5_)_3_ was used.

h The reaction time is 36 hours.

With improved reaction conditions in hand, the scope of the boron-catalysed cross coupling reaction was explored between a set of di-aryl allylic alcohols and boronic acids. At first, the substituent effects on the benzene ring of 1 was evaluated. Delightfully, both electric-donating and -withdrawing groups were well tolerated with the conditions. When methyl substituted allylic alcohol on the para-position was tested, 58% yield was obtained (3b). Products with halogen groups such as fluorine, chlorine and bromine were also generated in the yields up to 90% (3c–3e). Methyl, methoxyl and chlorine groups on meta and ortho positions of the benzene ring were also appropriate under standard conditions offering 3f to 3i in the yields of 23–78%. When the benzene ring was replaced with 2-naphthalene, the corresponding product was prepared in 45% yield (3j). Next, the scope of boronic acids were also explored. Apart from 2a, phenylboronic acid bearing a series of electron-rich groups such as alkoxyl, methylthio and dimethylamino groups were compatible with this protocol affording corresponding products 3k–3o in the yields of 45–90%. Further evaluation employing alkyl substituent allylic alcohol such as (*E*)pent-3-en-2-ol failed to yield the desired product. Later, several aryboronic acids including (4-methoxynaphthalen-1-yl) boronic acid, indole-5-boronic acid, benzofuran-3-boronic acid and benzofuran-2-boronic acid provided the desired products 3p–3s in 58–92% yields. Moreover, alkenyl boric acid was also evaluated offering 3t in the yield of 84%. It is always challenging to control the regio-selectivity of the nucleophilic substitution for unsymmetrical allylic alcohols.^9^ When electron deficient boronic acids such as (perfluorophenyl)boronic acid and pyridine heterocycle boronic acid were tested under the optimized conditions, the reaction did not occur with the boronic acids recovered. The regio-selectivity of this kind of reaction is later, to illustrate the regio-selectivity of the protocol further, the unsymmetrical allylic alcohol 1u was loaded to the standard conditions, and 3u was obtained as the only products in 85% yield, which emphasize the excellent selectivity of the reaction (Table 2).

**Table 2:** Scope of the reaction^a^

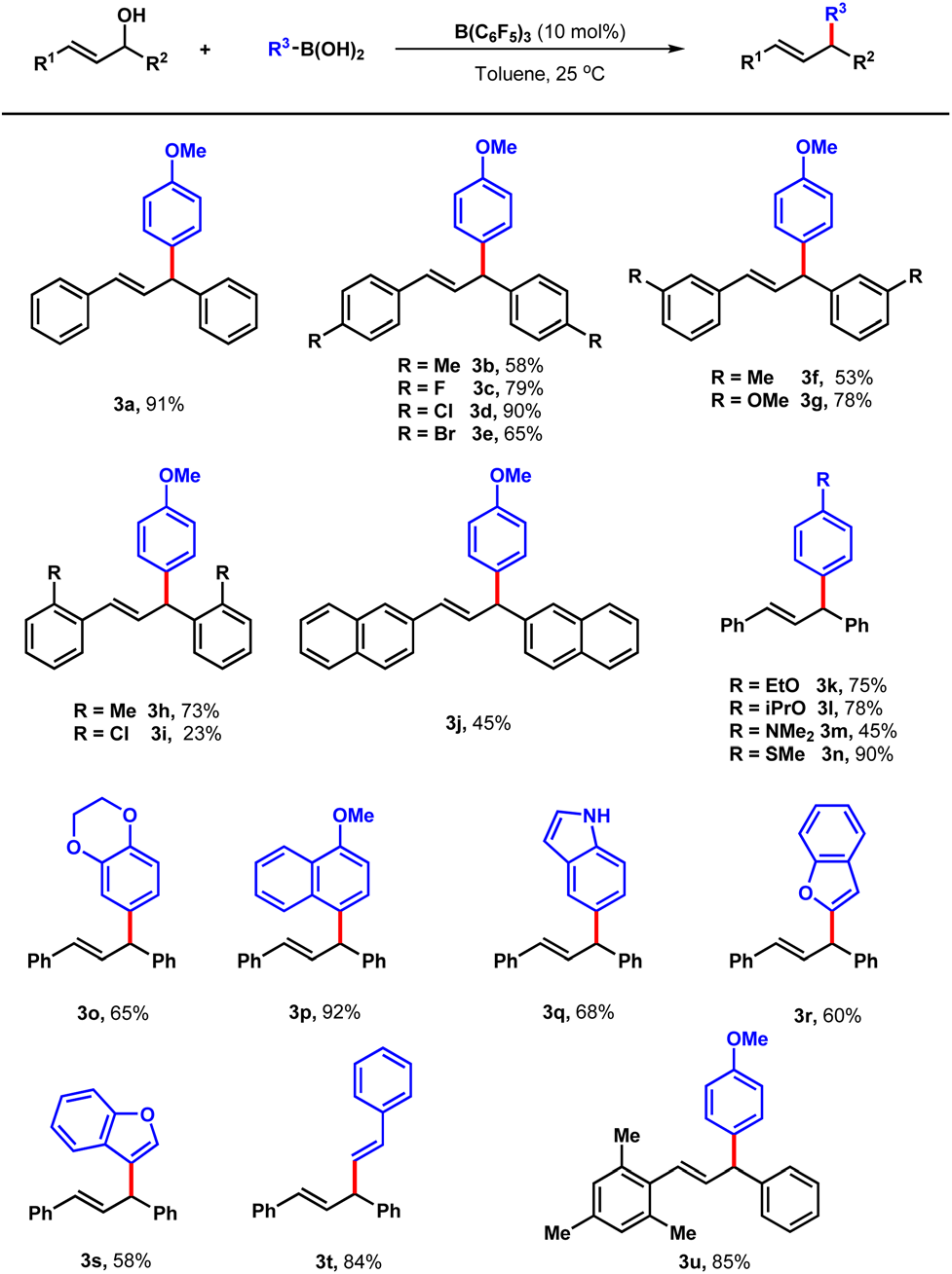

a Reaction conditions: 1 (0.1 mmol), 2 (0.2 mmol), B(C_6_F_5_)_3_ (10 mol%), toluene (2 mL), 36 hours, under air atmosphere.

Based on previous reports,^8^ the reaction mechanism was proposed as shown in Scheme 2. At first, the coordination of the B(C_6_F_5_)_3_ catalyst with the alcohol generated the intermediate a (Int a), which tend to offer the carbocation intermediate b (Int b) after the cleavage of C–O bond. Later, the migration of *R*^3^ to C1 or C3 position occurred to generate the desired product 3 and boric acid with the B(C_6_F_5_)_3_ catalyst recovered. The selectivity between the C1 or C3 arylation mainly depends on the steric effect of *R*^1^ and *R*^2^.

**Scheme 2:**
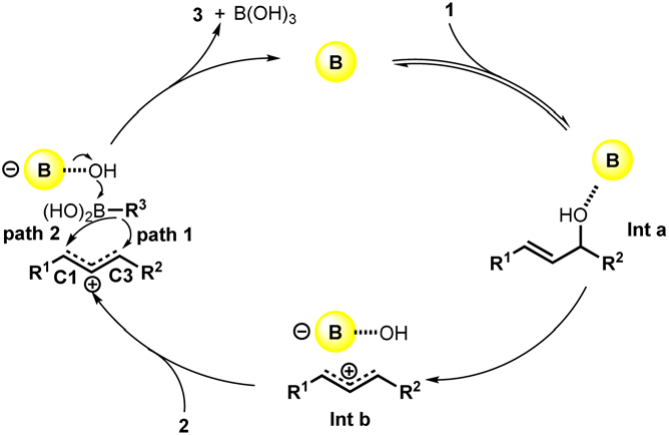
Proposed reaction pathway.

## Conclusions

In conclusion, we have established an efficient and convenient cross-coupling reaction between allylic alcohols and boronic acids under transition-metal-free conditions. This boronic-catalysed protocol exhibited excellent selectivity, mild conditions and broad scope.

## Conflicts of interest

There are no conflicts to declare.

## Supplementary Material

RA-013-D2RA07919D-s001
