# Supplementary material for: Boron-catalysed transition-metal-free arylation and alkenylation of allylic alcohols with boronic acids
Source: RSC Adv. 2023 Jan 23;13(5):3329–32. doi: 10.1039/d2ra07919d (PMC9869934; doi:10.1039/d2ra07919d)
Supplement: RA-013-D2RA07919D-s001 [file RA-013-D2RA07919D-s001.pdf]

This journal is © The Royal Society of Chemistry 2023

1. General information.

Reagents and Solvents: PE refers to petroleum ether (b.p. 60-90 °C), EA refers to ethyl acetate, MeCN refers to acetonitrile, Tf_2O refers to Trifluoromethanesulfonic anhydride, TFA refers to trifluoroacetic acid, $B(C_6F_5)_3$ refers to tris(pentafluorophenyl)borate, MeOH refers to methanol, EtOH refers to ethanol, $NaBH_4$ refers to sodium borohydride, $CeCl_3{\cdot}7H_2O$ refers to cerium(III) chloride heptahydrate. Unless noted, commercially available reagents were used without further purification.

Chromatography: Analytical thin layer chromatography (TLC) plates and the silica gel (200 - 300 mesh) for column chromatography were phased from Qingdao Haiyang Chemical and Special Silica Gel Co, Ltd. Gradient flash chromatography was conducted eluting with PE/EA which are listed as volume/volume ratios.

Data collection: ^1H and ^{13}C NMR spectra were collected on BRUKER AV-600 (600 MHz) spectrometer using $CDCl_3$ as solvent. Chemical shifts of ^1H NMR were recorded in parts per million (ppm, δ) relative to tetramethylsilane (δ = 0.00 ppm) with the solvent resonance as the internal standard ($CDCl_3$: δ = 7.26 ppm). Data are reported as follows: chemical shift in ppm (δ), multiplicity (s = singlet, d = doublet, t = triplet, q = quartet, m = multiplet), coupling constant (Hz), and integration. Chemical shifts of ^{13}C NMR were reported in ppm with the solvent as the internal standard. High Resolution Mass measurement was performed on Waters Q-TOF 6520 mass spectrometer with atmosphere pressure chemical ionization (APCI) as the ion source.

2. Preparation of Substrates

2.1 Involved substrates

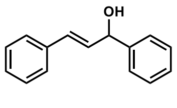

1a

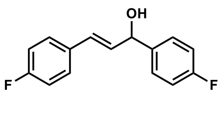

1b

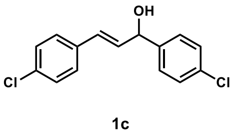

1c

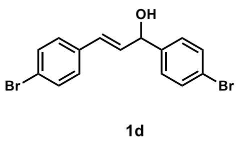

1d

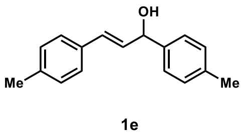

1e

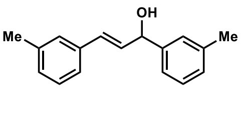

1f

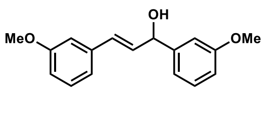

1g

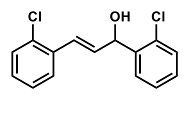

1h

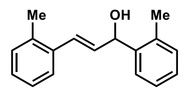

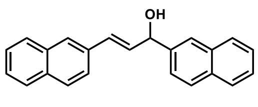

2.2 General procedure for the synthesis of alcohols 1

(*E*)-1,3-Diphenylprop-2-en-1-ol substrates were prepared according to the previously reported literature[1].

A typical procedure is shown as follow:

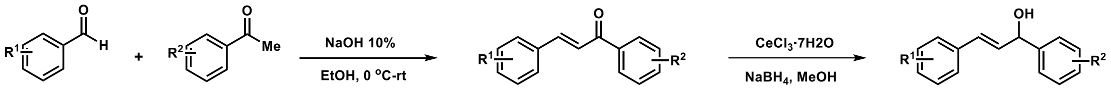

General procedure A: To a stirred solution of substituted acetophenone (10 mmol) in ethanol (30 mL), a aquous solution of 10% NaOH (12 mL) was added dropwise at 0 °C over a period of 10 min. After the addition is completed, the reaction mixture was stirred at 0 °C for 20 minutes then at room temprature for 1 hours. The resulting mixture was further treated with substituted benzaldehydes (2.5 mmol) and allowed to stirred at room temperature, until the conversion was complete (disappearance of acetophenone, monitored by TLC). The solvent was removed by evaporation and the residue was treated with water (10 mL) and extracted with ethyl acetate (30 mL × 3). The combined organic layer was dried over anhydrous sodium sulfate,concentrated, and purified through silica-gel column chromatography using ethyl acetate/petroleum ether as eluent to obtain the product α, β-unsaturated ketones (A) (chalcones, 68−95%). The compounds were reported in literature.

General procedure B: To a stirred solution of $CeCl_3·7H_2O$ (0.670 g, 1.8 mmol) and **A** (0.312 g, 1.5 mmol) in MeOH (10 mL) at 0 °C added sodium borohydride (45 mg, 1.8 mmol) was added portion wise and the reaction mixture was further stirred for 15 min at rt. Then, the reaction mixture was adjusted to pH 7 using a 10% HCl solution and extracted three times with EA. The combined organic layers were washed with brine and dried over anhydrous Na_2SO_4. Then the solvent was removed under reduced pressure and the crude residue was purified by column chromatography on silica-gel using ethyl acetate/petroleum ether as eluent to obtain the product **1**. The compounds were reported in literature.

3. Experimental procedure for the synthesis

3.1 Procedure for gram scale synthesis

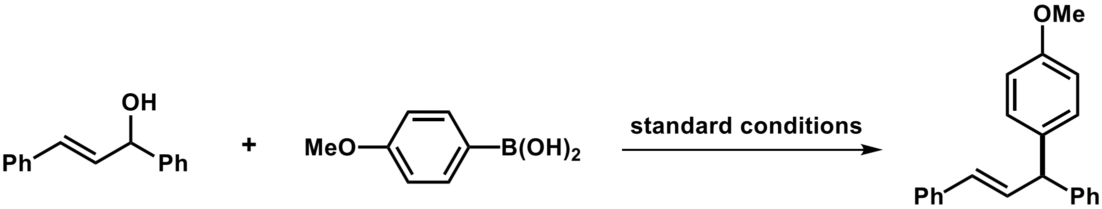

1a, 5 mmol 2a, 10 mmol 3a, 1.24 g, 82%

Cinnamyl alcohol **1a** (5 mmol, 1.05 g), **2a** (10 mmol, 1.51 g) and $B(C_6F_5)_3$ (10 mol%, 255 mg) in toluene (20 mL) was taken in a round bottomed flask (50 mL) under the air atmosphere and the reaction mixture was stirred for 36 h at rt. The completion of the reaction was monitored by TLC or GC-MS analysis. The reaction mixture was extracted with ethyl acetate (30 mL × 3). The combined organic layers were washed with brine and dried over anhydrous sodium sulfate, concentrated, and purified through silica-gel column chromatography using petroleum ether and ethyl acetate as eluent (97:3).

3.2 Substitution position

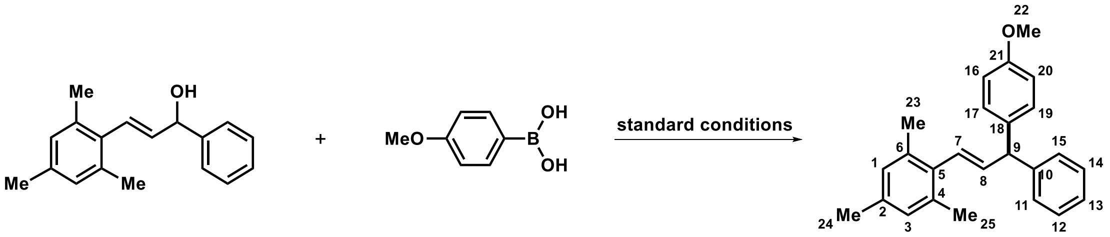

From the ^{1}H-NMR ($CDCl_3$, 600 MHz) spectrum, the two hydrogen signals [δ 6.27 (d, *J* = 16.1 Hz, 1H), 6.11 (dd, *J* = 16.1, 7.4 Hz, 1H)] were typical hydrogen signal characteristics of alkene H-7, H-8. The HMBC spectrum showed that δ H 6.27 (d, *J* = 16.1 Hz) was remotely correlated with δ C 135.8 (C-6), δ C 135.8 (C-4) and δ C 53.9 (C-9). From the HSQC spectrum, there was no hydrogen signal directly correlating with C-6, C-4. The ^{1}H-NMR and ^{13}C-NMR spectral signals combined with the ^{1}H-^{1}H COSY, HSQC, and HMBC spectral information were inferred that substitution position was C-9.

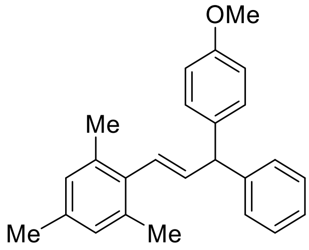

Transparent oil; ^{1}H NMR (600 MHz, $CDCl_3$) δ 7.33 – 7.28 (m, 2H), 7.25 (d, *J* = 8.2 Hz, 2H), 7.23 – 7.19 (m, 1H), 7.16 (d, *J* = 8.7 Hz, 2H), 6.85 (d, *J* = 8.8 Hz, 2H), 6.84 (s, 2H), 6.27 (d, *J* = 16.1 Hz, 1H), 6.11 (dd, *J* = 16.1, 7.4 Hz, 1H), 4.88 (d, *J* = 7.4 Hz, 1H), 3.79 (s, 3H), 2.25 (s, 3H), 2.23 (s, 6H) ppm; ^{13}C NMR (150 MHz, $CDCl_3$) δ 158.1, 144.1, 137.4, 135.9, 135.9, 135.8, 134.2, 129.5, 129.1, 128.6, 128.5, 128.4, 126.3, 113.8, 55.2, 53.9, 21.0, 20.9 ppm.

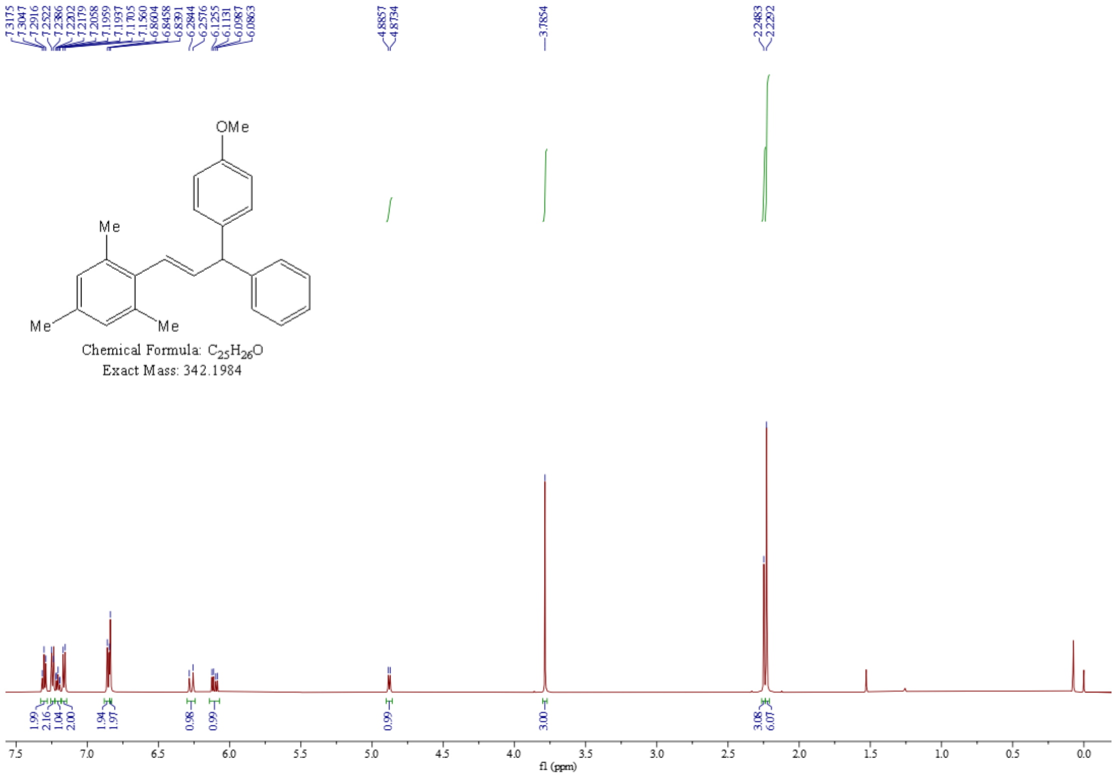
OMe
Me
Me
Me
Chemical Formula: $C_{25}H_{26}O$
Exact Mass: 342.1984
f1 (ppm)

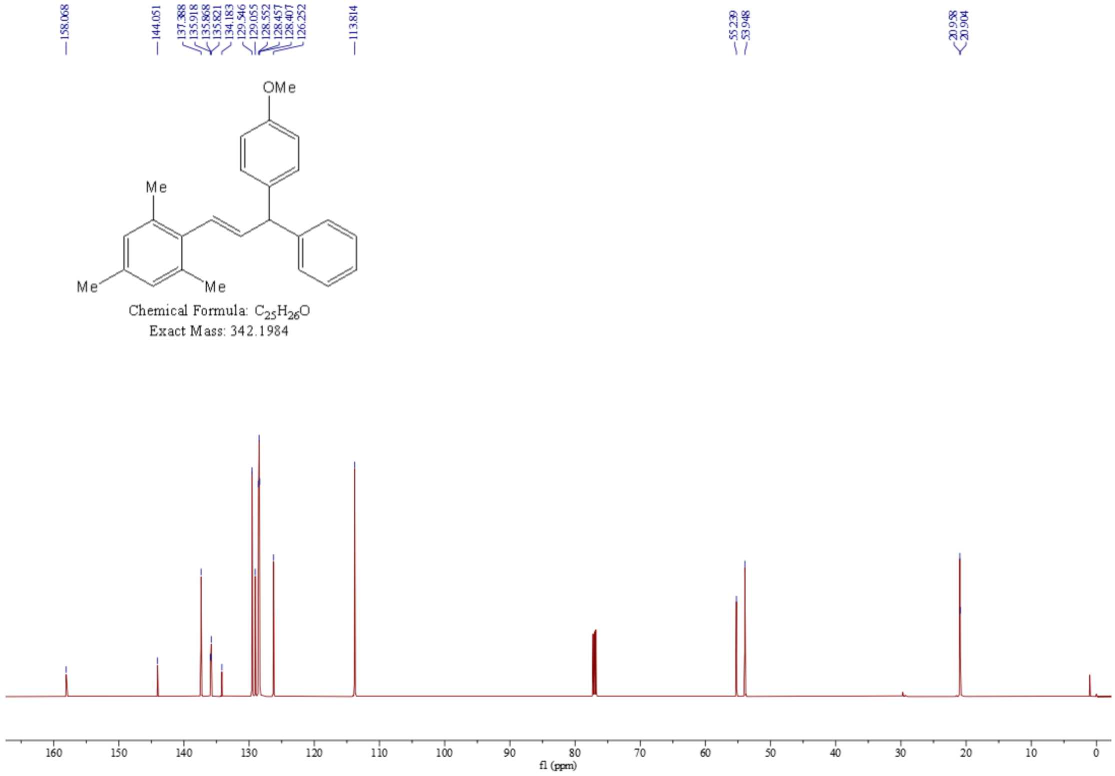
OMe
Me
Me
Me
Chemical Formula: $C_{25}H_{26}O$
Exact Mass: 342.1984
f1 (ppm)

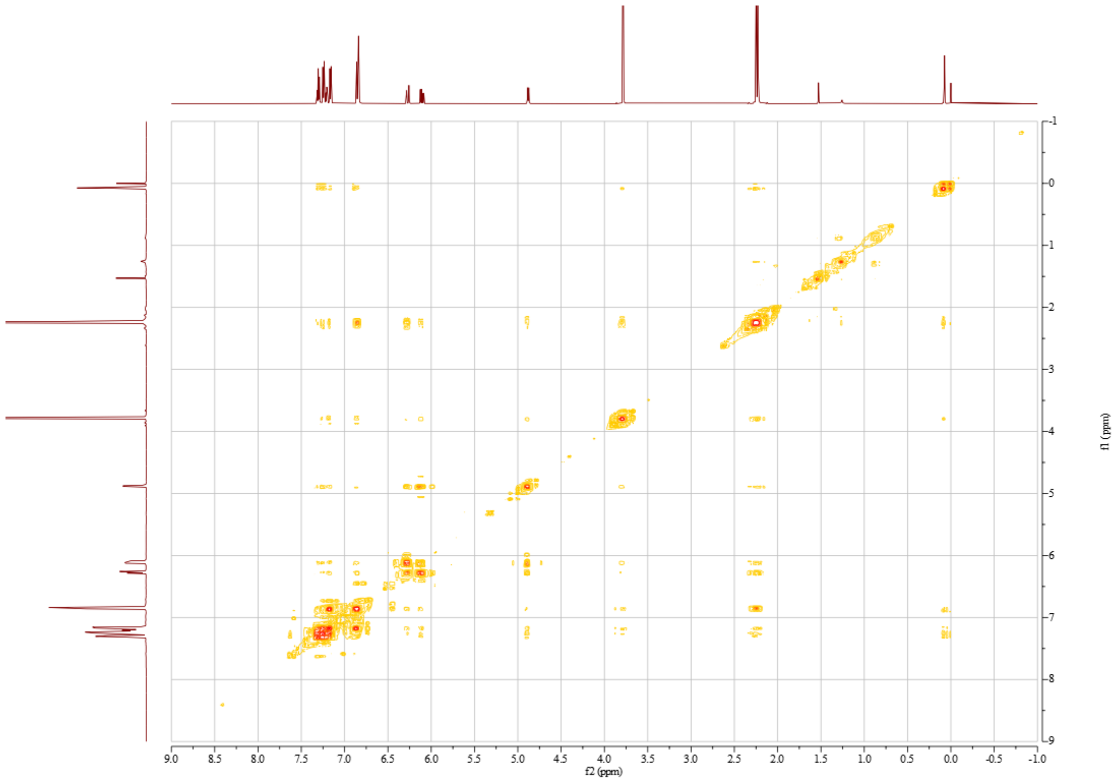

COSY

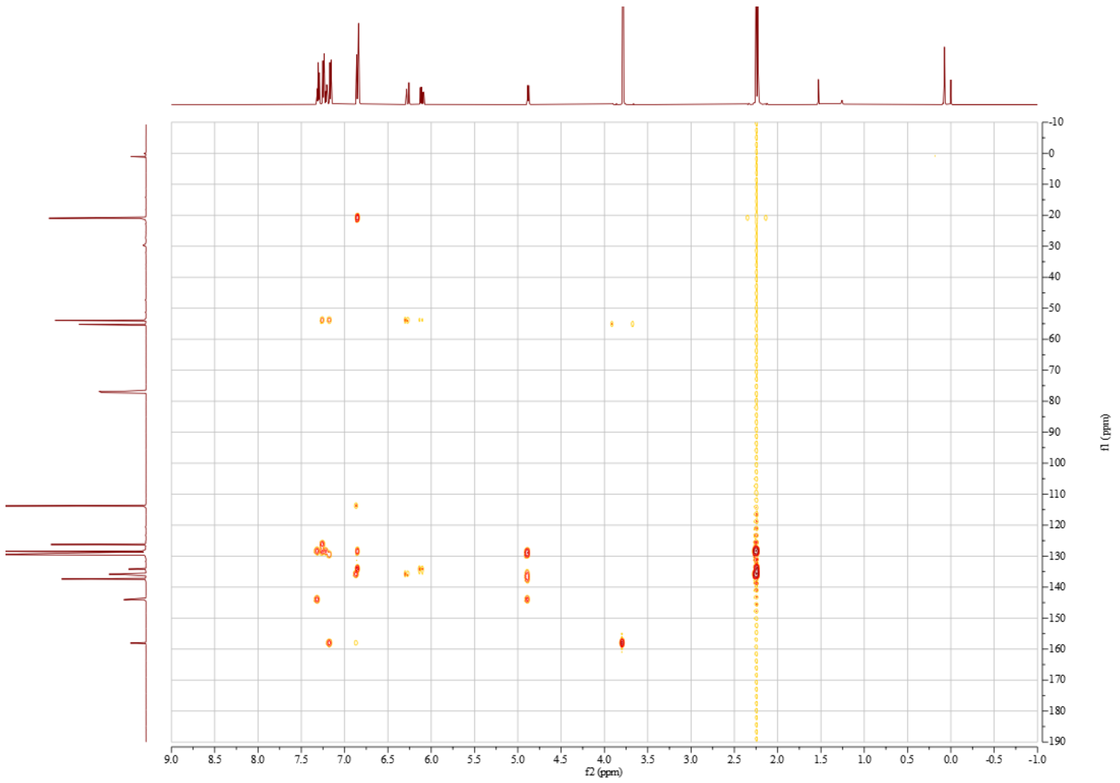

HMBC

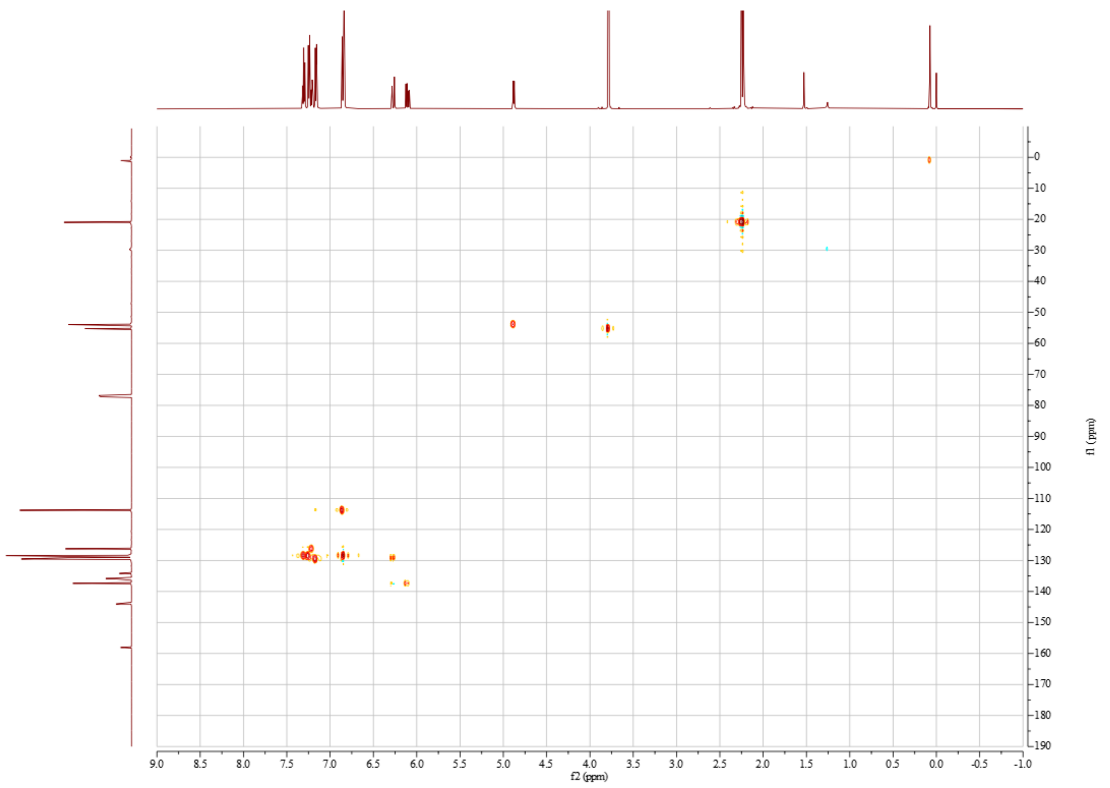

HSQC

Detail descriptions for products

(E)-(3-(4-methoxyphenyl)prop-1-ene-1,3-diyl)dibenzene (3a)

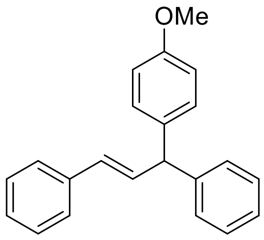

Yield 91%; Colorless oil; ^{1}H NMR (600 MHz, $CDCl_3$) δ 7.37 (d, J = 6.9 Hz, 2H), 7.33 – 7.27 (m, 4H), 7.25 – 7.19 (m, 4H), 7.15 (d, J = 8.6 Hz, 2H), 6.85 (d, J = 8.7 Hz, 2H), 6.65 (dd, J = 15.8, 7.5 Hz, 1H), 6.33 (d, J = 15.9 Hz, 1H), 4.85 (d, J = 7.5 Hz, 1H), 3.79 (s, 3H) ppm; ^{13}C NMR (150 MHz, $CDCl_3$) δ 158.2, 143.8, 137.4, 135.7, 132.9, 131.2, 129.6, 128.6, 128.5, 128.5, 127.3, 126.4, 126.3, 113.9, 55.3, 53.4 ppm; GC-MS (EI) calcd for [$C_{22}H_{20}O$] 300, found 300.

(E)-4,4'-(3-(4-methoxyphenyl)prop-1-ene-1,3-diyl)bis(methylbenzene) (3b)

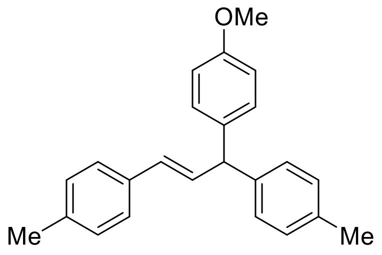

Yield 58%; Colorless oil; ^{1}H NMR (600 MHz, $CDCl_3$) δ 7.26 – 7.25 (m, 2H), 7.15 – 7.08 (m, 8H), 6.84 (d, J = 8.7 Hz, 2H), 6.58 (dd, J = 15.8, 7.5 Hz, 1H), 6.28 (d, J = 15.9 Hz, 1H), 4.79 (d, J = 8.6 Hz, 1H), 3.78 (s, 3H), 2.32 (d, J = 3.5 Hz, 6H) ppm; ^{13}C NMR (150 MHz, $CDCl_3$) δ 158.0, 141.0, 136.9, 136.0, 135.8, 134.6, 132.1, 130.8, 129.5, 129.2, 129.1, 128.5, 126.2, 113.8, 55.2, 52.9, 21.1, 21.0 ppm; GC-MS (EI) calcd for [$C_{24}H_{24}O$] 328, found 328.

(E)-1,3-bis(4-fluorophenyl)prop-2-en-1-ol (3c)

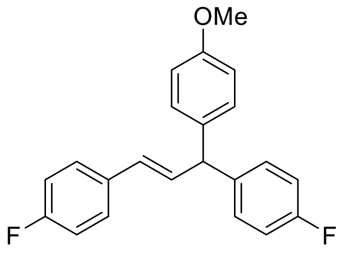

Yield 79%; Colorless oil; ^{1}H NMR (600 MHz, $CDCl_3$) δ 7.31 (dd, *J* = 8.6, 5.5 Hz, 2H), 7.16 (dd, *J* = 8.5, 5.5 Hz, 2H), 7.11 (d, *J* = 8.6 Hz, 2H), 6.98 (q, *J* = 8.6 Hz, 4H), 6.85 (d, *J* = 8.7 Hz, 2H), 6.51 (dd, *J* = 15.8, 7.4 Hz, 1H), 6.25 (d, *J* = 15.8 Hz, 1H), 4.81 (d, *J* = 7.3 Hz, 1H), 3.79 (s, 3H) ppm; ^{13}C NMR (150 MHz, $CDCl_3$) 158.3, 139.4, 139.4, 135.3, 133.3, 133.3, 132.4, 132.4, 130.1, 130.0, 129.9, 129.5, 127.8, 127.7, 115.5, 115.3, 115.3, 115.2, 113.9, 55.3, 52.5 ppm; ^{19}F NMR (400 MHz, $CDCl_3$) δ -114.73 (s, 1F), -116.65 (s, 1F); GC-MS (EI) calcd for [$C_{22}H_{18}F_2O$] 336, found 336.

(E) -4,4'-(3-(4-methoxyphenyl)prop-1-ene-1,3-diyl)bis(chlorobenzene) (3d)

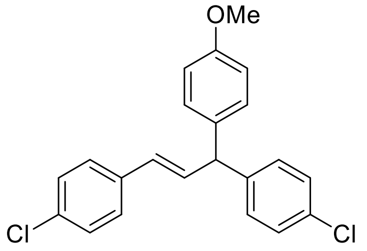

Yield 90%; Colorless oil; ^{1}H NMR (600 MHz, $CDCl_3$) δ 7.28 – 7.24 (m, 6H), 7.12 (dd, *J* = 20.0, 8.5 Hz, 4H), 6.85 (d, *J* = 8.7 Hz, 2H), 6.56 (dd, *J* = 15.8, 7.4 Hz, 1H), 6.24 (d, *J* = 15.8 Hz, 1H), 4.80 (d, *J* = 7.3 Hz, 1H), 3.79 (s, 3H) ppm; ^{13}C NMR (150 MHz, $CDCl_3$) δ 158.3, 142.0, 135.6, 134.8, 133.0, 132.3, 130.3, 129.9, 129.5, 128.7, 128.6, 127.5, 114.0, 55.3, 52.6 ppm; GC-MS (EI) calcd for [$C_{22}H_{18}Cl_2O$] 368, found 368.

(F) -4,4'-(3-(4-methoxyphenyl)prop-1-ene-1,3-diyl)bis(bromobenzene) (3e)

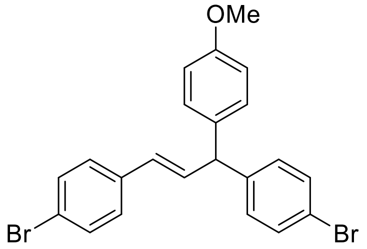

Yield 65%; Colorless oil; ^{1}H NMR (600 MHz, $CDCl_3$) δ 7.46 – 7.36 (m, 4H), 7.21 (d, *J* = 8.5 Hz, 2H), 7.08 (dd, *J* = 11.8, 8.6 Hz, 4H), 6.85 (d, *J* = 8.7 Hz, 2H), 6.57 (dd, *J* = 15.8, 7.3 Hz, 1H), 6.22 (d, *J* = 14.4 Hz, 1H), 4.78 (d, *J* = 7.3 Hz, 1H), 3.78 (s, 3H) ppm; ^{13}C NMR (150 MHz, $CDCl_3$) δ 158.4, 142.5, 136.0, 134.6, 133.0, 131.6, 131.6, 130.4, 130.3, 129.5, 127.8, 121.1, 120.4, 114.0, 55.3, 52.7 ppm; GC-MS (EI) calcd for [$C_{22}H_{18}Br_2O$] 458, found 458.

(E)-3,3'-(3-(4-methoxyphenyl)prop-1-ene-1,3-diyl)bis(methylbenzene) (3f)

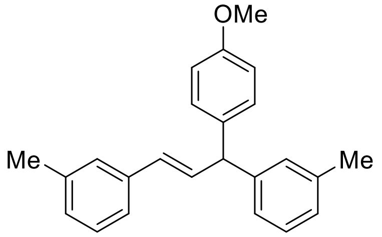

Yield 53%; Colorless oil; ^{1}H NMR (600 MHz, $CDCl_3$) δ 7.21 – 7.17 (m, 4H), 7.14 (d, *J* = 8.6 Hz, 2H), 7.03 (d, *J* = 13.6 Hz, 4H), 6.85 (d, *J* = 8.7 Hz, 2H), 6.63 (dd, *J* = 15.8, 7.5 Hz, 1H), 6.29 (d, *J* = 15.9 Hz, 1H), 4.80 (d, *J* = 7.5 Hz, 1H), 3.79 (s, 3H), 2.32 (d, *J* = 2.1 Hz, 6H) ppm; ^{13}C NMR (150 MHz, $CDCl_3$) δ 158.1, 143.8, 138.0, 137.3, 135.8, 132.8, 131.1, 129.6, 129.3, 128.4, 128.3, 128.0, 127.1, 127.0, 125.6, 123.4, 113.8, 55.2, 53.3, 21.5, 21.4 ppm; GC-MS (EI) calcd for [$C_{24}H_{24}O$] 328, found 328.

(E)-3,3'-(3-(4-methoxyphenyl)prop-1-ene-1,3-diyl)bis(methoxybenzene) (3g)

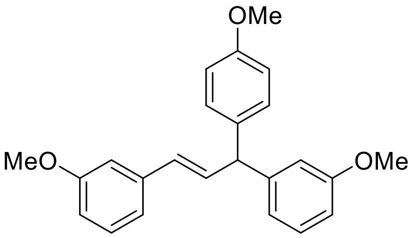

Yield 78%; Pale yellow oil; ^{1}H NMR (600 MHz, $CDCl_3$) δ 7.25 – 7.20 (m, 2H), 7.15 (d, *J* = 8.6 Hz, 2H), 6.96 (d, *J* = 7.8 Hz, 1H), 6.91 – 6.88 (m, 1H), 6.85 (d, *J* = 8.7 Hz, 2H), 6.82 (d, *J* = 7.7 Hz, 1H), 6.76 (dd, *J* = 11.1, 2.9 Hz, 3H), 6.62 (dd, *J* = 15.8, 7.5 Hz, 1H), 6.30 (d, *J* = 15.7 Hz, 1H), 4.81 (d, *J* = 7.4 Hz, 1H), 3.79 (d, *J* = 3.0 Hz, 6H), 3.76 (s, 3H) ppm; ^{13}C NMR (150 MHz, $CDCl_3$) δ 159.8, 159.7, 158.2, 145.4, 138.8, 135.4, 133.0, 131.1, 129.6, 129.4, 129.4, 121.0, 119.0, 114.6, 113.8, 113.0, 111.5, 111.4, 55.2, 55.2, 55.2, 53.3 ppm; GC-MS (EI) calcd for [$C_{24}H_{24}O_3$] 360, found 360.

(E)-2,2'-(3-(4-methoxyphenyl)prop-1-ene-1,3-diyl)bis(methylbenzene) (3h)

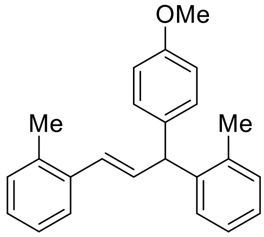

Yield 73%; Colorless oil; ^{1}H NMR (600 MHz, $CDCl_3$) δ 7.47 (d, *J* = 7.1 Hz, 1H), 7.21 – 7.08 (m, 9H), 6.84 (d, *J* = 8.7 Hz, 2H), 6.49 (dd, *J* = 15.7, 6.7 Hz, 1H), 6.41 (d, *J* = 15.7 Hz, 1H), 5.04 (d, *J* = 6.6 Hz, 1H), 3.79 (s, 3H), 2.30 (s, 3H), 2.22 (s, 3H) ppm; ^{13}C NMR (150 MHz, $CDCl_3$) δ 158.0, 141.9, 136.6, 136.3, 135.2, 135.0, 134.2, 130.5, 130.1, 129.8, 129.1, 128.4, 127.1, 126.4, 126.0, 125.6, 113.7, 55.2, 49.8, 19.7 ppm; GC-MS (EI) calcd for [$C_{24}H_{24}O$] 328, found 328.

(E)-2,2'-(3-(4-methoxyphenyl)prop-1-ene-1,3-diyl)bis(chlorobenzene) (3i)

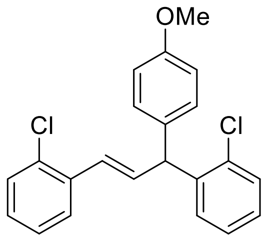

Yield 23%; Colorless oil; ^{1}H NMR (600 MHz, $CDCl_3$) δ 7.55 (dd, *J* = 7.7, 1.7 Hz, 1H), 7.38 (d, *J* = 7.9 Hz, 1H), 7.32 (d, *J* = 7.9 Hz, 1H), 7.25 – 7.13 (m, 7H), 6.86 (d, *J* = 8.7 Hz, 2H), 6.68 (d, *J* = 15.8 Hz, 1H), 6.57 (dd, *J* = 15.9, 6.9 Hz, 1H), 5.37 (d, *J* = 6.9 Hz, 1H), 3.79 (s, 3H) ppm; ^{13}C NMR (150 MHz, $CDCl_3$) δ 158.3, 140.9, 135.5, 134.4, 134.2, 133.8, 133.0, 130.0, 129.8, 129.7, 129.6, 128.3, 128.3, 127.8, 126.9, 126.9, 126.7, 113.9, 55.2, 49.6 ppm; GC-MS (EI) calcd for [$C_{22}H_{18}Cl_2O$] 368, found 368.

(E)-2,2'-(3-(4-methoxyphenyl)prop-1-ene-1,3-diyl)dinaphthalene (3j)

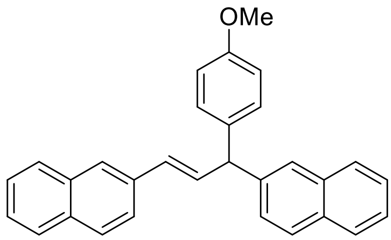

Yield 45%; Colorless oil; ^{1}H NMR (600 MHz, $CDCl_3$) δ 7.82 – 7.74 (m, 6H), 7.69 (d, *J* = 8.2 Hz, 2H), 7.63 (d, *J* = 8.6 Hz, 1H), 7.46 – 7.36 (m, 5H), 7.22 (d, *J* = 8.7 Hz, 2H), 6.86 (dd, *J* = 15.5, 7.9 Hz, 3H), 6.52 (d, *J* = 15.8 Hz, 1H), 5.07 (d, *J* = 7.3 Hz, 1H), 3.80 (s, 3H) ppm; ^{13}C NMR (150 MHz, $CDCl_3$) δ 158.2, 141.3, 135.4, 134.7, 133.6, 133.5, 133.1, 132.8, 132.2, 131.6, 129.8, 128.1, 128.1, 127.9, 127.8, 127.6, 127.6, 127.4, 126.8, 126.2, 126.1, 126.0, 125.7, 125.6, 123.6, 113.9, 55.3, 53.5 ppm; GC-MS (EI) calcd for [$C_{30}H_{24}O$] 400, found 400.

(E)-(3-(4-ethoxyphenyl)prop-1-ene-1,3-diyl)dibenzene (3k)

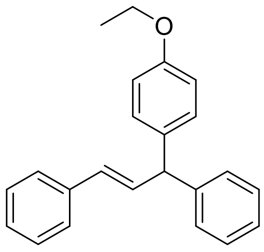

Yield 75%; Colorless oil; ^{1}H NMR (600 MHz, $CDCl_3$) δ 7.37 (d, *J* = 8.1 Hz, 2H), 7.30 (dt, *J* = 12.6, 7.5 Hz, 4H), 7.26 – 7.20 (m, 4H), 7.14 (d, *J* = 8.6 Hz, 2H), 6.85 (d, *J* = 8.7 Hz, 2H), 6.66 (dd, *J* = 15.8, 7.5 Hz, 1H), 6.33 (d, *J* = 17.1 Hz, 1H), 4.85 (d, *J* = 7.5 Hz, 1H), 4.02 (q, *J* = 7.0 Hz, 2H), 1.41 (t, *J* = 7.0 Hz, 3H) ppm; ^{13}C NMR (150 MHz, $CDCl_3$) δ 157.5, 143.9, 137.3, 135.5, 132.9, 131.1, 129.6, 128.6, 128.5, 128.4, 127.2, 126.4, 126.3, 114.4, 63.4, 53.3, 14.9 ppm; GC-MS (EI) calcd for [$C_{23}H_{22}O$] 314, found 314.

(E)-(3-(4-isopropoxyphenyl)prop-1-ene-1,3-diyl)dibenzene (3l)

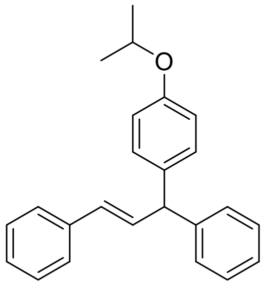

Yield 78%; Colorless oil; ^{1}H NMR (600 MHz, $CDCl_3$) δ 7.36 (d, *J* = 6.8 Hz, 2H), 7.29 (dt, *J* = 12.9, 7.5 Hz, 4H), 7.25 – 7.20 (m, 4H), 7.12 (d, *J* = 8.7 Hz, 2H), 6.83 (d, *J* = 6.7 Hz, 2H), 6.65 (dd, *J* = 15.8, 7.5 Hz, 1H), 6.33 (d, *J* = 15.6 Hz, 1H), 4.83 (d, *J* = 7.5 Hz, 1H), 4.51 (p, *J* = 6.0 Hz, 1H), 1.32 (d, *J* = 6.1 Hz, 6H) ppm; ^{13}C NMR (150 MHz, $CDCl_3$) δ 156.4, 143.8, 137.3, 135.4, 133.0, 131.1, 129.6, 128.6, 128.5, 128.4, 127.2, 126.3, 126.3, 115.7, 69.8, 53.4, 22.1 ppm; GC-MS (EI) calcd for [$C_{24}H_{24}O$] 328, found 328.

(E)-4-(1,3-diphenylallyl)-N,N-dimethylaniline (3m)

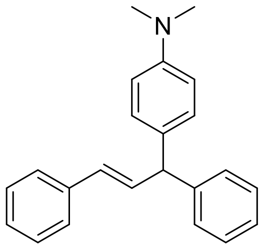

Yield 45%; Colorless oil; ^{1}H NMR (600 MHz, $CDCl_3$) δ 7.37 (d, *J* = 8.1 Hz, 2H), 7.31 – 7.24 (m, 6H), 7.20 (d, *J* = 6.2 Hz, 2H), 7.11 (d, *J* = 8.4 Hz, 2H), 6.71 (d, *J* = 8.3 Hz, 2H), 6.67 (dd, *J* = 15.3, 7.0 Hz, 1H), 6.34 (d, *J* = 15.8 Hz, 1H), 4.82 (d, *J* = 7.5 Hz, 1H), 2.93 (s, 6H) ppm; ^{13}C NMR (150 MHz, $CDCl_3$) δ 149.2, 144.2, 137.5, 133.3, 131.5, 130.8, 129.3, 128.6, 128.5, 128.4, 127.1, 126.3, 126.2, 112.8, 53.3, 40.7 ppm; GC-MS (EI) calcd for [$C_{23}H_{23}N$] 313, found 313.

(E)-(4-(1,3-diphenylallyl)phenyl)(methyl)sulfane (3n)

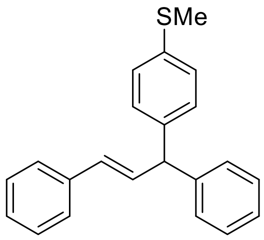

Yield 90%; Colorless oil; ^{1}H NMR (600 MHz, $CDCl_3$) δ 7.38 (d, *J* = 6.9 Hz, 2H), 7.31 (dt, *J* = 13.1, 7.6 Hz, 4H), 7.25 – 7.21 (m, 6H), 7.17 (d, *J* = 8.3 Hz, 2H), 6.65 (dd, *J* = 15.8, 7.5 Hz, 1H), 6.35 (d, *J* = 14.5 Hz, 1H), 4.86 (d, *J* = 7.5 Hz, 1H), 2.47 (s, 3H) ppm; ^{13}C NMR (150 MHz, $CDCl_3$) δ 143.4, 140.5, 137.2, 136.3, 132.4, 131.5, 129.2, 128.6, 128.5, 127.4, 126.9, 126.5, 126.3, 53.6, 16.0 ppm; GC-MS (EI) calcd for [$C_{22}H_{20}S$] 316, found 316.

(E)-6-(1,3-diphenylallyl)-2,3-dihydrobenzo[b][1,4]dioxine (3o)

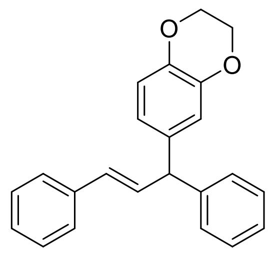

Yield 65%; Colorless oil; ^{1}H NMR (600 MHz, $CDCl_3$) δ 7.36 (d, *J* = 6.9 Hz, 2H), 7.29 (dt, *J* = 12.8, 7.5 Hz, 4H), 7.25 – 7.20 (m, 4H), 6.80 (d, *J* = 8.3 Hz, 1H), 6.74 (d, *J* = 2.1 Hz, 1H), 6.70 (dd, *J* = 8.3, 2.2 Hz, 1H), 6.62 (dd, *J* = 15.8, 7.6 Hz, 1H), 6.34 (d, *J* = 14.5 Hz, 1H), 4.78 (d, J = 7.6 Hz, 1H), 4.23 (s, 4H) ppm; ^{13}C NMR (150 MHz, $CDCl_3$) δ 143.6, 143.3, 142.1, 137.3, 136.9, 132.6, 131.2, 128.5, 128.5, 128.5, 127.2, 126.4, 126.3, 121.6, 117.3, 117.1, 64.4, 64.3, 53.5 ppm; GC-MS (EI) calcd for [$C_{23}H_{20}O_2$] 328, found 328.

(E)-1-(1,3-diphenylallyl)-4-methoxynaphthalene (3p)

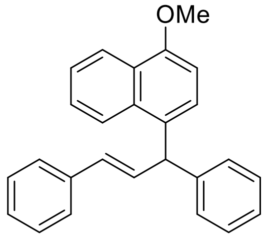

Yield 92%; Colorless oil; ^{1}H NMR (600 MHz, $CDCl_3$) δ 8.37 – 8.30 (m, 1H), 8.01 – 7.96 (m, 1H), 7.48 – 7.43 (m, 2H), 7.37 (d, *J* = 6.9 Hz, 2H), 7.33 – 7.26 (m, 7H), 7.25 – 7.19 (m, 2H), 6.84 – 6.77 (m, 2H), 6.26 (d, *J* = 17.5 Hz, 1H), 5.58 (d, *J* = 8.1 Hz, 1H), 4.00 (s, 3H) ppm; ^{13}C NMR (150 MHz, $CDCl_3$) δ 154.6, 143.4, 137.4, 133.0, 132.6, 131.6, 131.1, 128.9, 128.5, 128.4, 127.2, 126.6, 126.5, 126.4, 126.3, 126.1, 124.9, 124.0, 122.5, 103.2, 55.5, 49.7 ppm; GC-MS (EI) calcd for [$C_{26}H_{22}O$] 350, found 350.

(E) -5-(1,3-diphenylallyl)-1H-indole (3q)

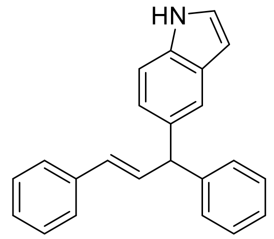

Yield 68%; Colorless oil; ^{1}H NMR (600 MHz, $CDCl_3$) δ 8.03 (s, 1H), 7.46 (dd, *J* = 8.0, 1.0 Hz, 1H), 7.41 – 7.36 (m, 5H), 7.36 – 7.30 (m, 4H), 7.28 – 7.18 (m, 3H), 7.06 (ddd, *J* = 8.0, 7.0, 1.0 Hz, 1H), 6.94 (dd, *J* = 2.5, 1.0 Hz, 1H), 6.76 (dd, *J* = 15.8, 7.4 Hz, 1H), 6.47 (dd, *J* = 15.8, 1.3 Hz, 1H), 5.16 (d, *J* = 7.4 Hz, 1H) ppm; ^{13}C NMR (150 MHz, $CDCl_3$) δ 143.4, 137.5, 136.7, 132.5, 130.6, 128.5, 128.5, 128.4, 127.2, 126.8, 126.4, 126.3, 122.6, 122.1, 119.9, 119.5, 118.8, 111.1, 46.2 ppm; GC-MS (EI) calcd for [$C_{23}H_{19}O$] 309, found 309.

(E)-2-(1,3-diphenylallyl)benzofuran (3r)

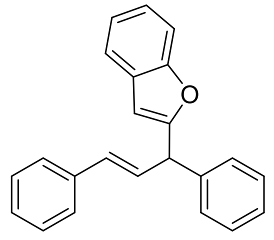

Yield 60%; Yellow oil; ^{1}H NMR (600 MHz, $CDCl_3$) δ 7.52 (d, *J* = 7.6 Hz, 1H), 7.44 (d, *J* = 7.6 Hz, 1H), 7.40 (d, *J* = 7.1 Hz, 2H), 7.37 – 7.33 (m, 4H), 7.33 – 7.28 (m, 3H), 7.26 – 7.19 (m, 3H), 6.66 (dd, *J* = 15.8, 7.5 Hz, 1H), 6.50 (d, *J* = 17.3 Hz, 2H), 5.05 (d, *J* = 7.5 Hz, 1H) ppm; ^{13}C NMR (150 MHz, $CDCl_3$) δ 159.3, 155.0, 140.4, 136.9, 132.2, 129.0, 128.7, 128.6, 128.5, 128.4, 127.6, 127.1, 126.4, 123.7, 122.6, 120.6, 111.1, 104.0, 48.7 ppm; GC-MS (EI) calcd for [$C_{23}H_{18}O$] 310, found 310.

(E)-3-(1,3-diphenylallyl)benzofuran (3s)

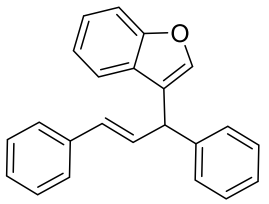

Yield 58%; Pale yellow oil; ^{1}H NMR (600 MHz, $CDCl_3$) δ 7.51 (dd, J = 7.0, 1.2 Hz, 1H), 7.43 (d, J = 7.6 Hz, 1H), 7.41 – 7.34 (m, 6H), 7.32 (d, J = 5.1 Hz, 1H), 7.30 (d, J = 7.8 Hz, 2H), 7.25 – 7.19 (m, 3H), 6.65 (dd, J = 15.8, 7.4 Hz, 1H), 6.49 (d, J = 18.5 Hz, 2H), 5.04 (d, J = 7.4 Hz, 1H) ppm; ^{13}C NMR (150 MHz, $CDCl_3$) δ 159.3, 155.0, 140.4, 136.9, 132.2, 129.0, 128.7, 128.6, 128.5, 128.4, 127.6, 127.1, 126.4, 123.7, 122.6, 120.6, 111.1, 104.0, 48.7 ppm; GC-MS (EI) calcd for [$C_{23}H_{18}O$] 310, found 310.

((1E,4E)-penta-1,4-diene-1,3,5-triyl)tribenzene (3t)

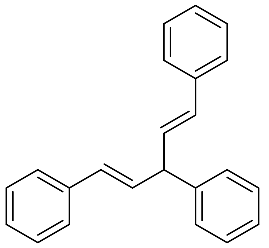

Yield 84%; White solid, m.p. 155-157 °C; ^{1}H NMR (600 MHz, $CDCl_3$) δ 7.39 (d, *J* = 6.8 Hz, 4H), 7.37 – 7.29 (m, 8H), 7.26 – 7.20 (m, 3H), 6.51 – 6.47 (m, 4H), 4.40 (s, 1H) ppm; ^{13}C NMR (150 MHz, $CDCl_3$) δ 142.8, 137.3, 131.9, 130.8, 128.6, 128.5, 128.1, 127.3, 126.6, 126.3, 51.6 ppm; GC-MS (EI) calcd for [$C_{23}H_{20}$] 296, found 296.

(R,E)-2-(3-(4-methoxyphenyl)-3-phenylprop-1-en-1-yl)-1,3,5-trimethylbenzene (3u)

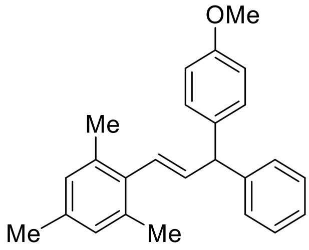

Yield 85%; Colorless oil; ^{1}H NMR (600 MHz, $CDCl_3$) δ 7.33 – 7.28 (m, 2H), 7.25 (d, *J* = 8.2 Hz, 2H), 7.23 – 7.19 (m, 1H), 7.16 (d, *J* = 8.7 Hz, 2H), 6.85 (d, *J* = 8.8 Hz, 2H), 6.84 (s, 2H), 6.27 (d, *J* = 16.1 Hz, 1H), 6.11 (dd, *J* = 16.1, 7.4 Hz, 1H), 4.88 (d, *J* = 7.4 Hz, 1H), 3.79 (s, 3H), 2.25 (s, 3H), 2.23 (s, 6H) ppm; ^{13}C NMR (150 MHz, $CDCl_3$) δ 158.1, 144.1, 137.4, 135.9, 135.9, 135.8, 134.2, 129.5, 129.1, 128.6, 128.5, 128.4, 126.3, 113.8, 55.2, 53.9, 21.0, 20.9 ppm.

^{1}H and ^{13}C-NMR Spectra of Title Compounds

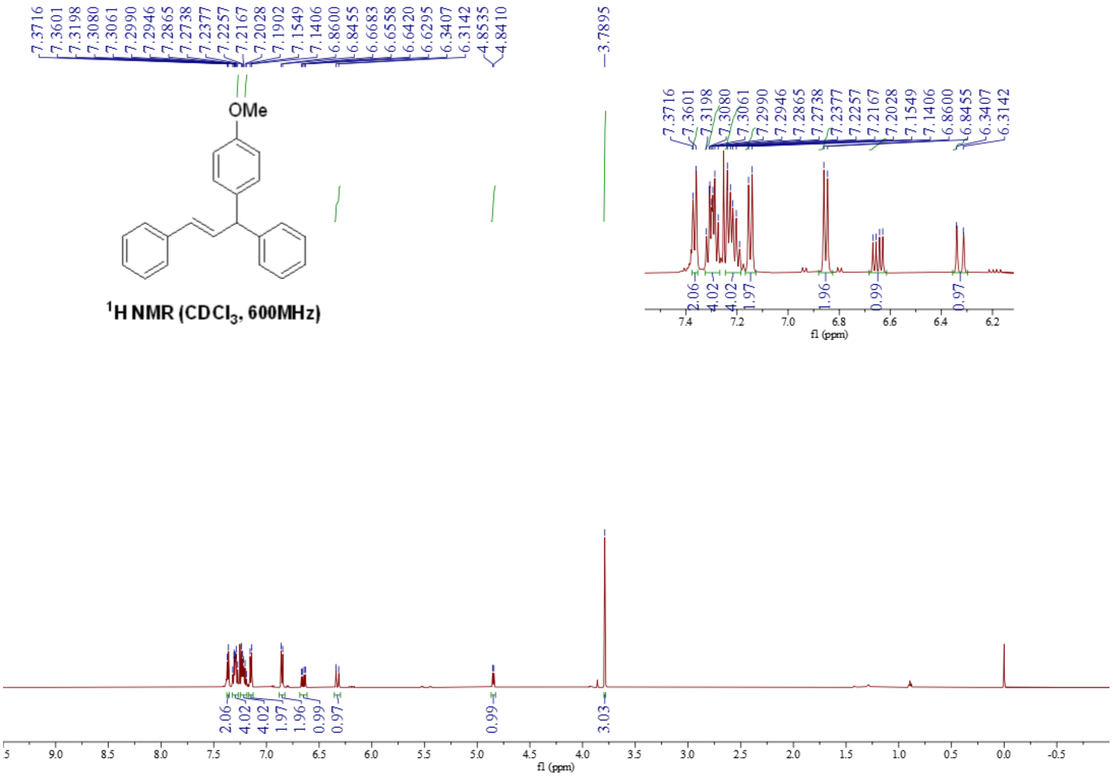

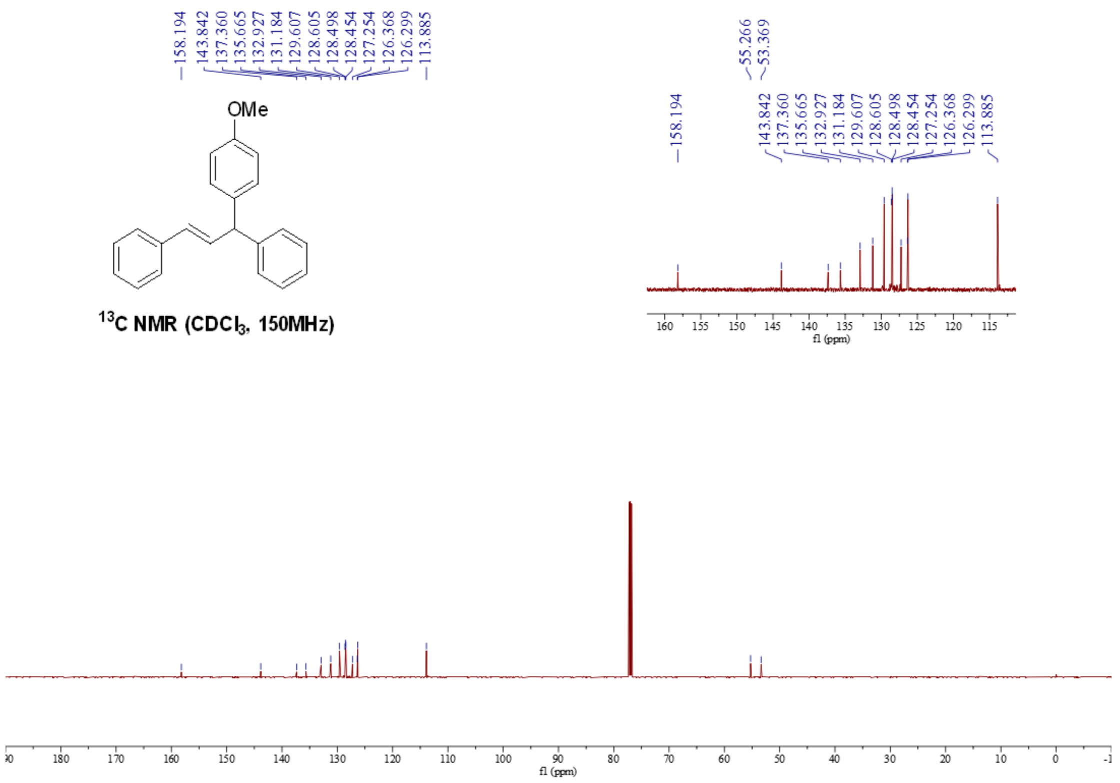

^{13}C NMR ($CDCl_3$, 150MHz)

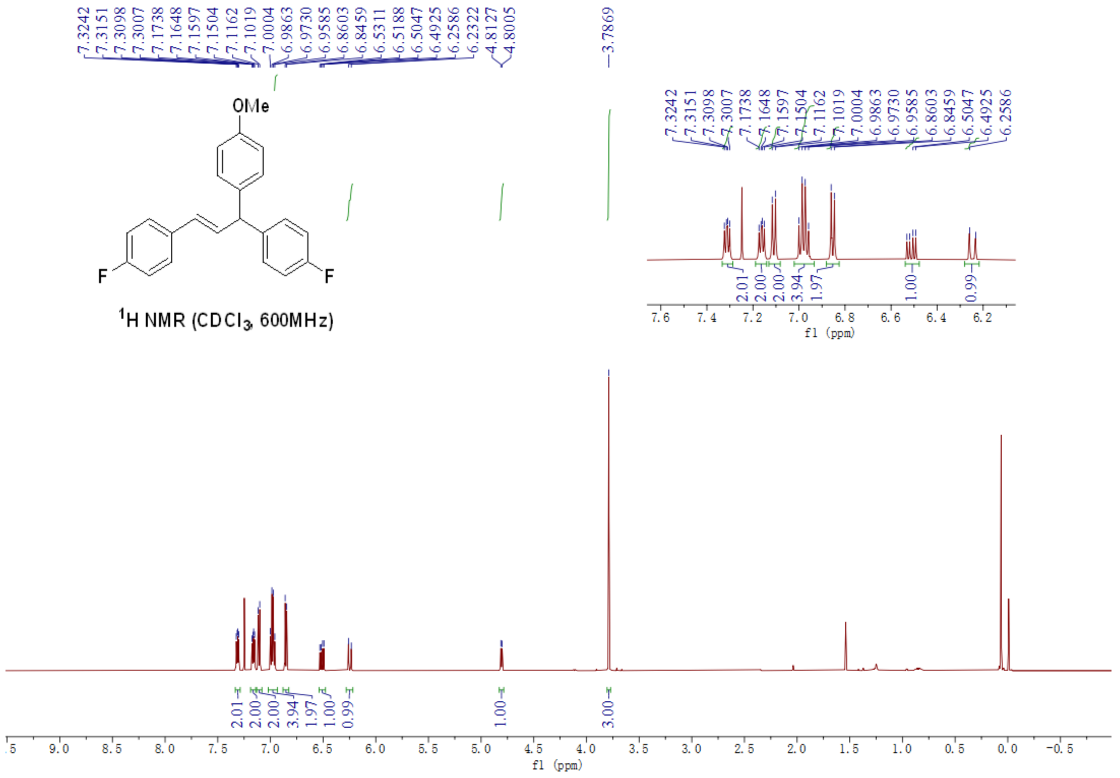

^{1}H NMR ($CDCl_3$, 600MHz)

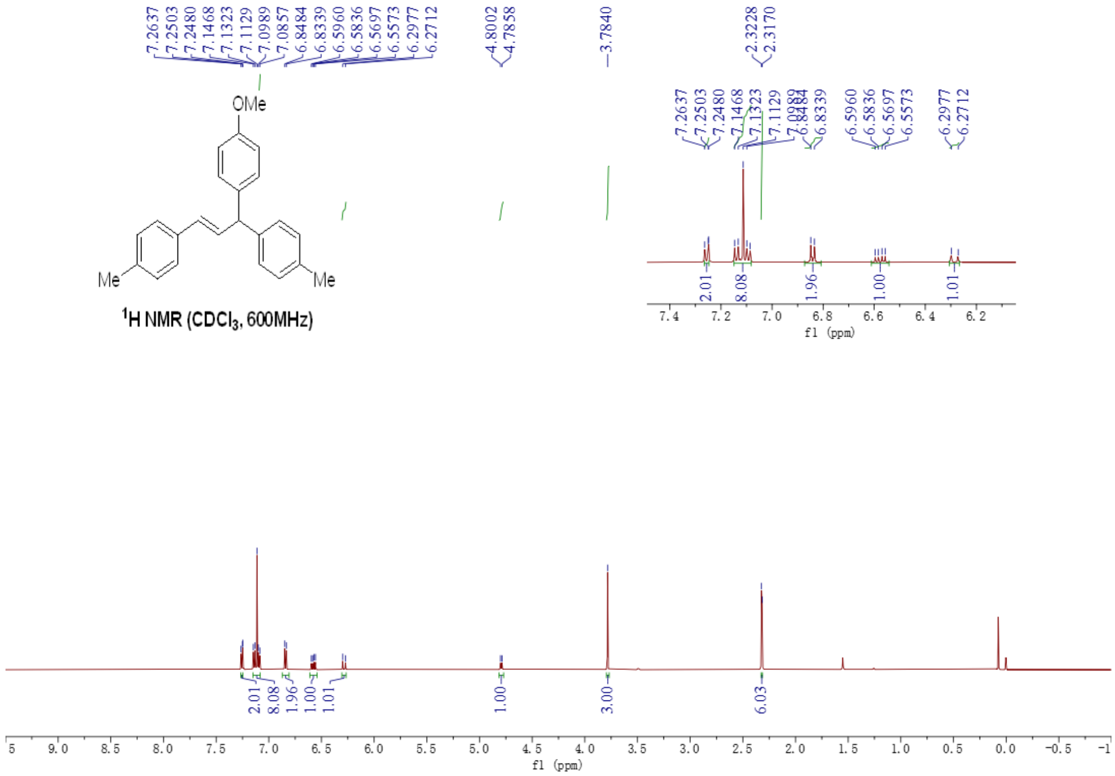

^{1}H NMR (CDCl$_3$, 600MHz)

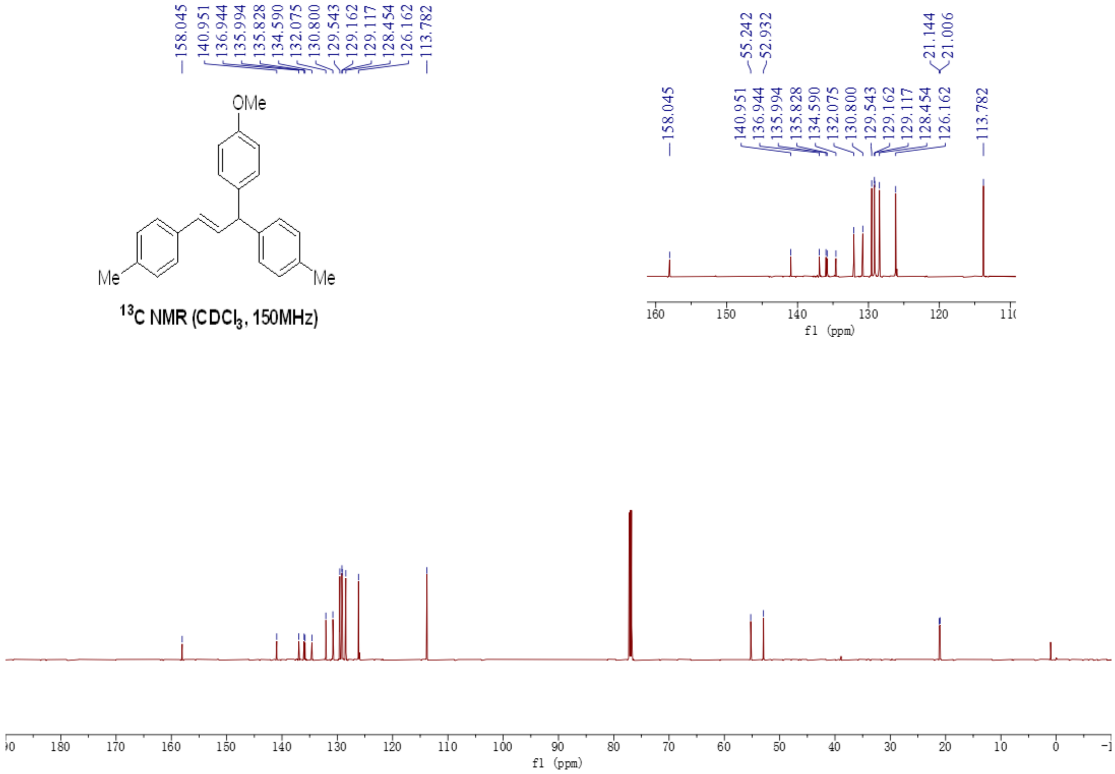

^{13}C NMR (CDCl$_3$, 150MHz)

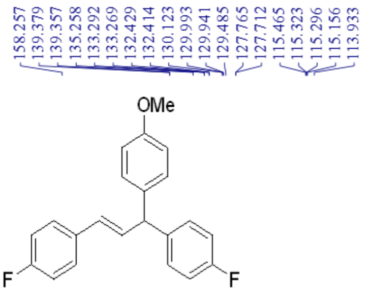

^{13}C NMR ($CDCl_3$, 150MHz)

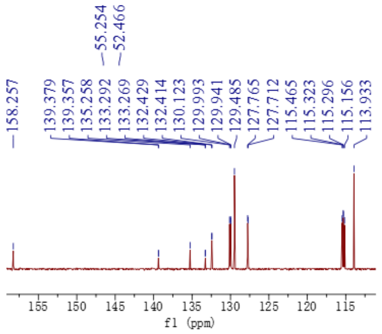

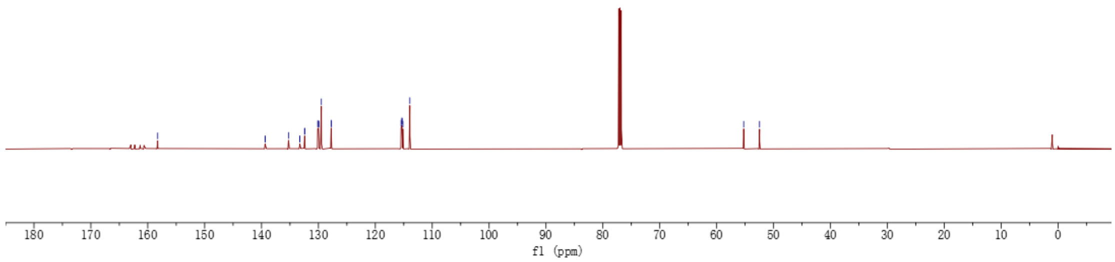

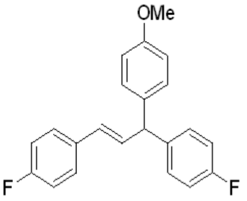

^{19}F NMR ($CDCl_3$, 400MHz)

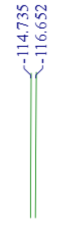

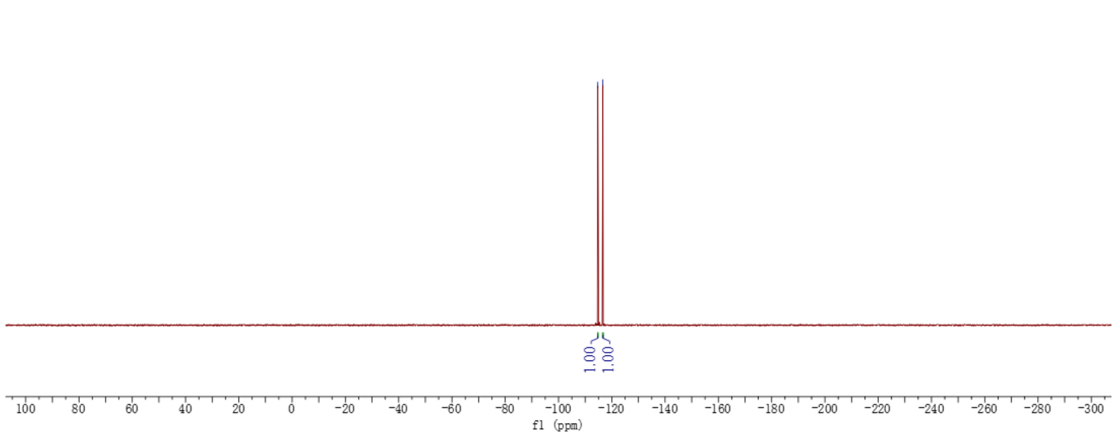

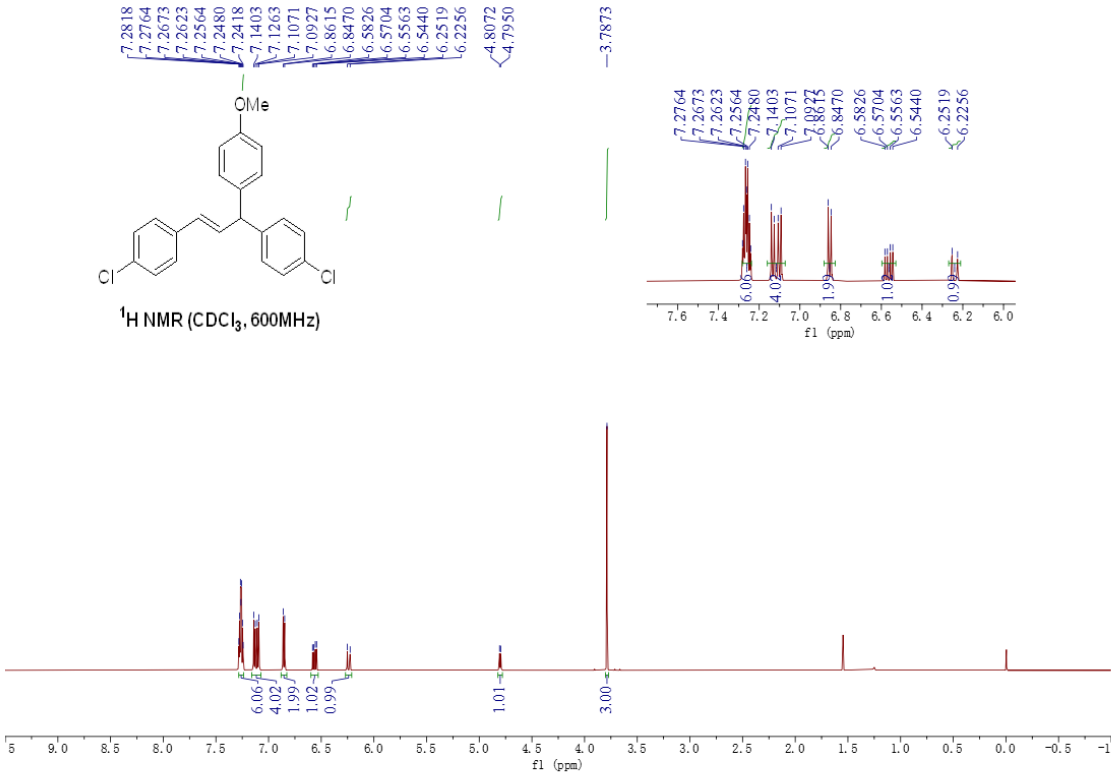

^{1}H NMR (CDCl$_3$, 600MHz)

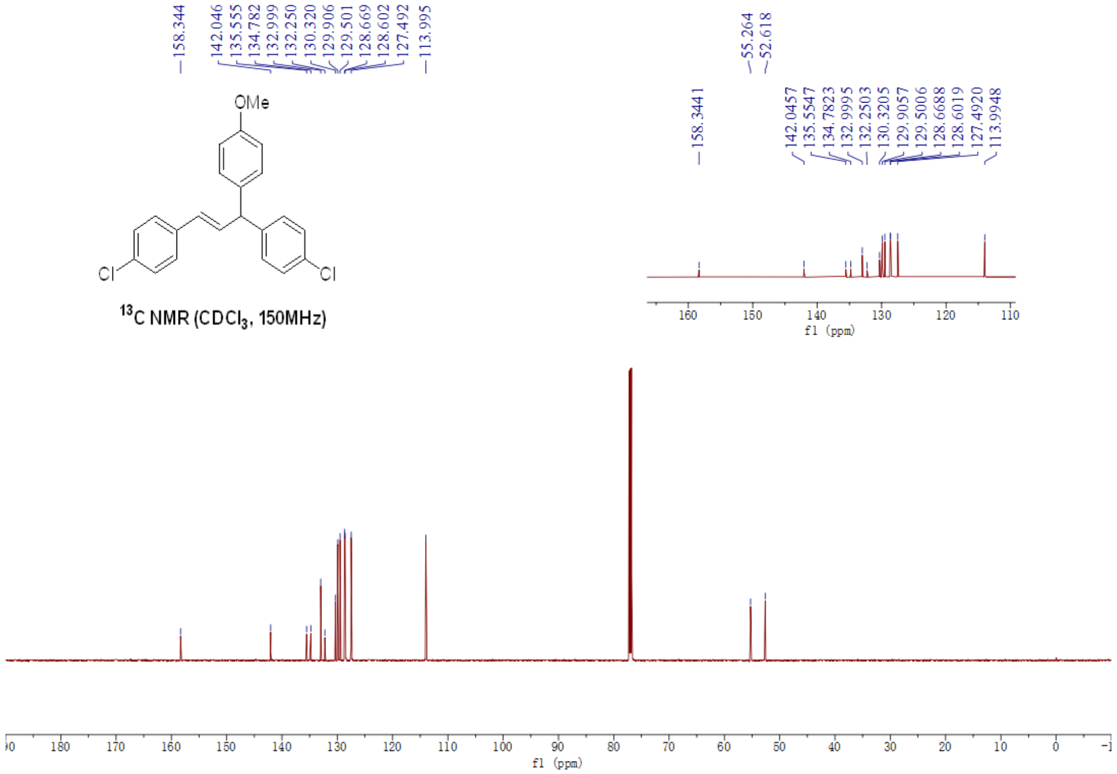

^{13}C NMR (CDCl$_3$, 150MHz)

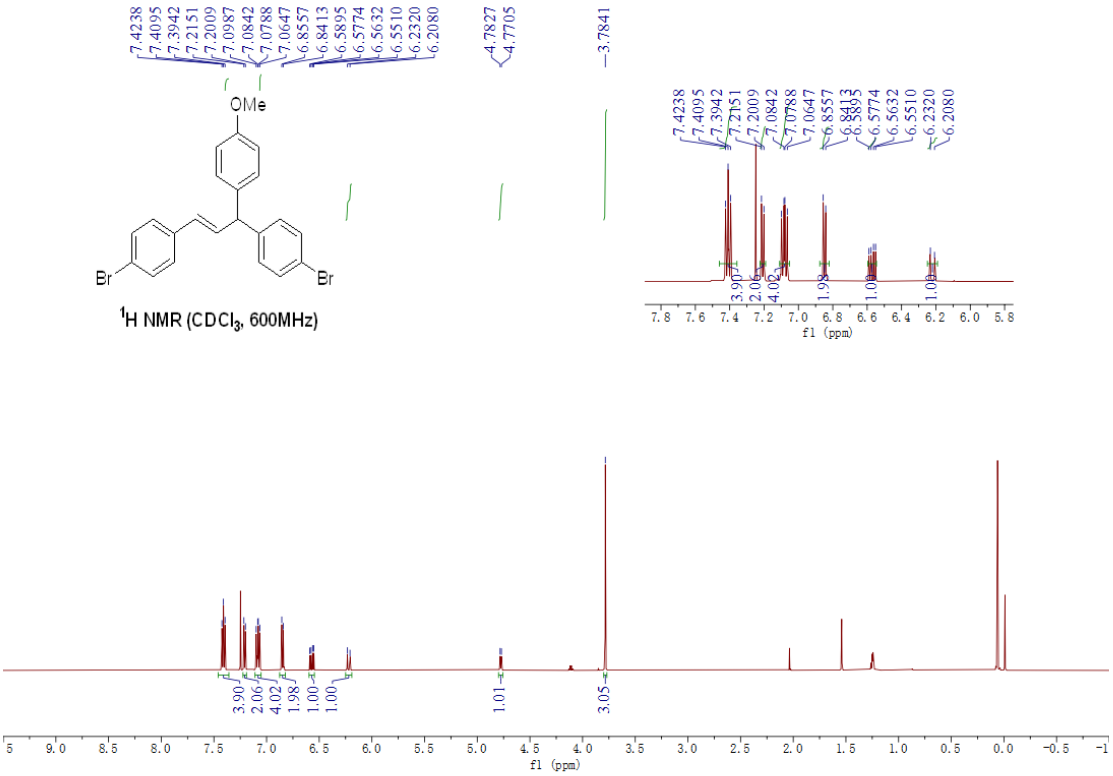

¹H NMR ($CDCl_3$, 600MHz)

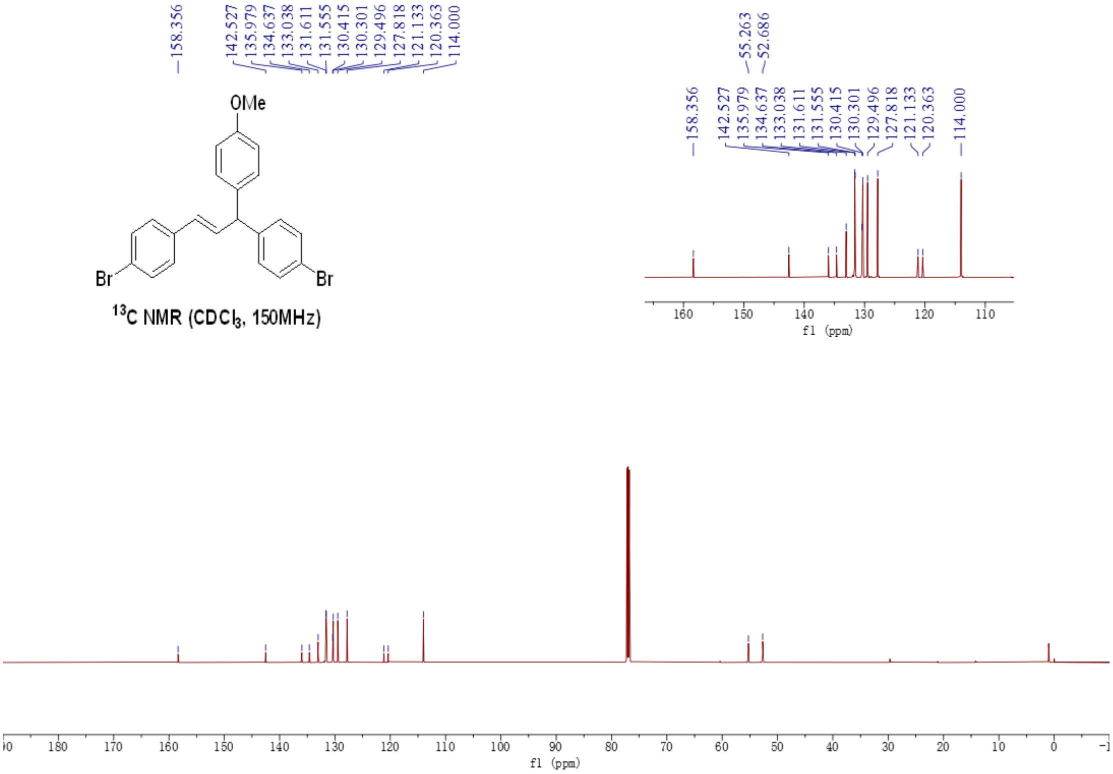

¹³C NMR ($CDCl_3$, 150MHz)

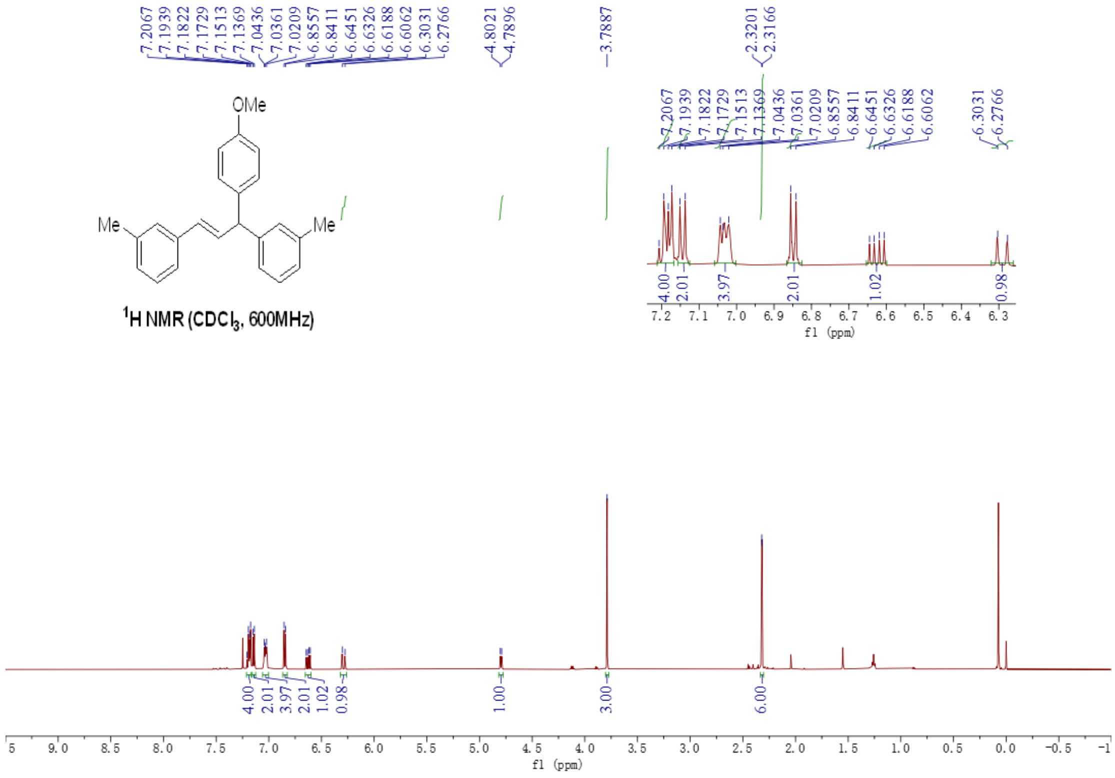
7.2067
7.1939
7.1822
7.1729
7.1513
7.1369
7.0436
7.0361
7.0209
6.8557
6.8411
6.6451
6.6326
6.6188
6.6062
6.3031
6.2766
4.8021
4.7896
3.7887
2.3201
2.3166
OMe
Me
Me
4.00
2.01
3.97
2.01
1.02
0.98
1.00
3.00
6.00
f1 (ppm)
1H NMR (CDCl3, 600MHz)

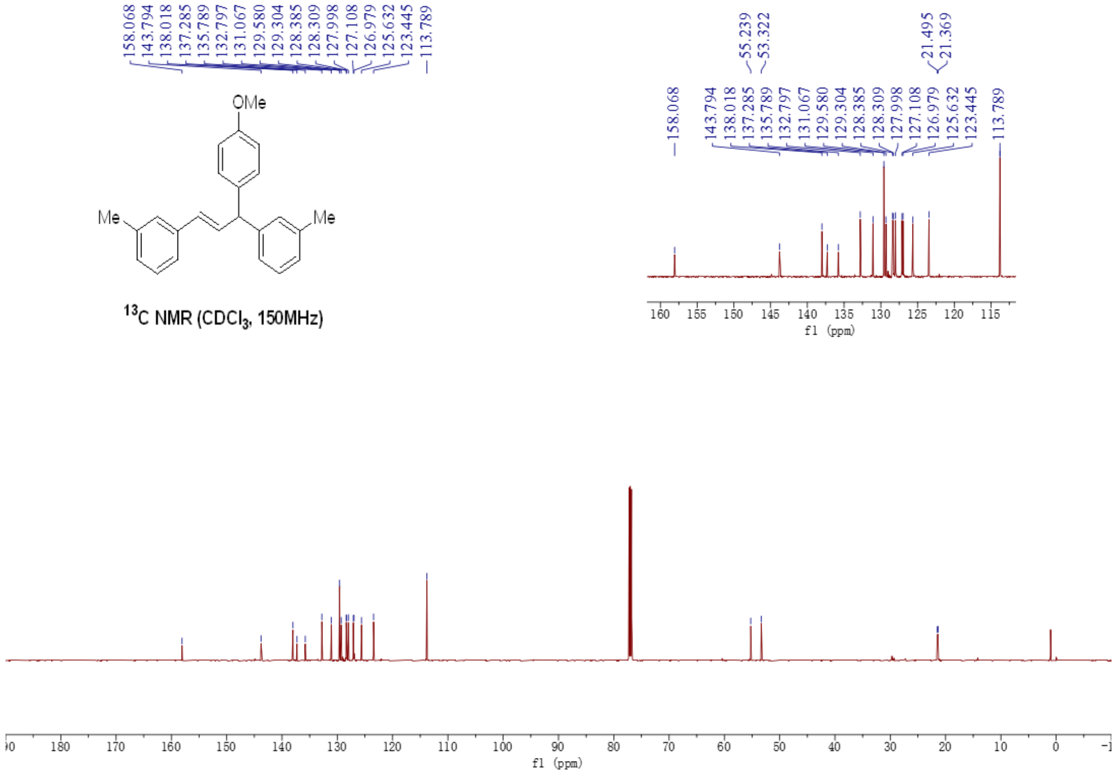
158.068
143.794
138.018
137.285
135.789
132.797
131.067
129.580
129.304
128.385
128.309
127.998
127.108
126.979
125.632
123.445
113.789
55.239
53.322
21.495
21.369
OMe
Me
Me
f1 (ppm)
13C NMR (CDCl3, 150MHz)

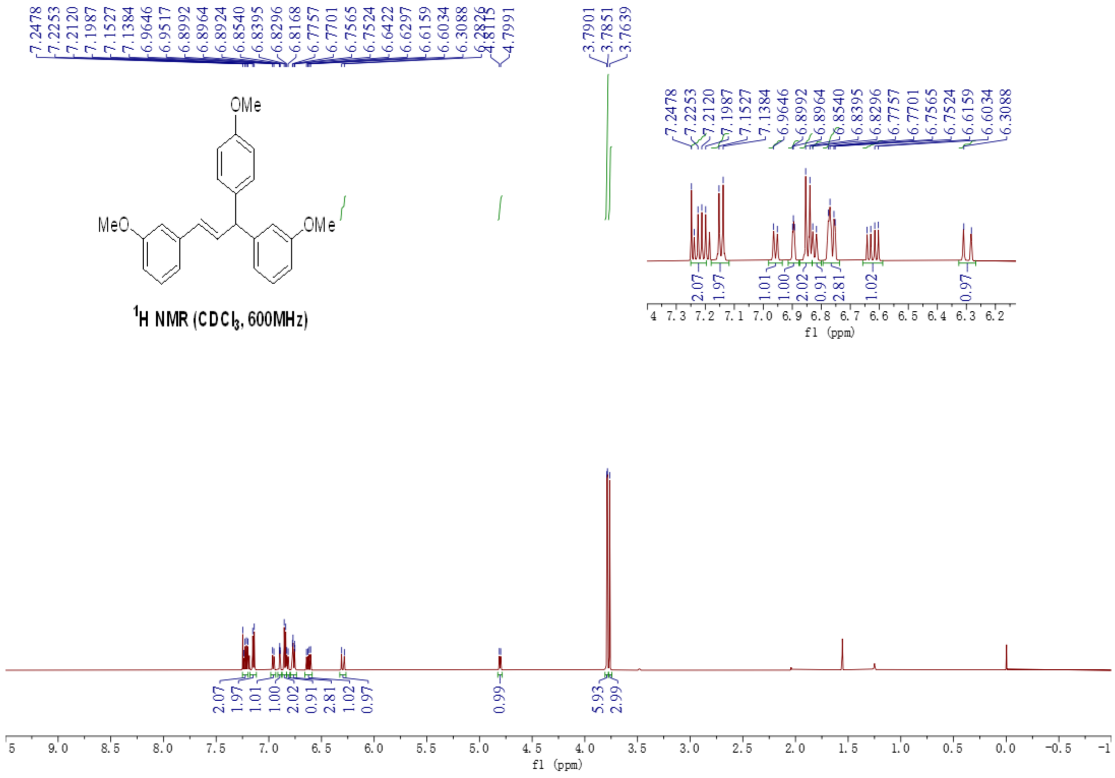

^{1}H NMR ($CDCl_3$, 600MHz)

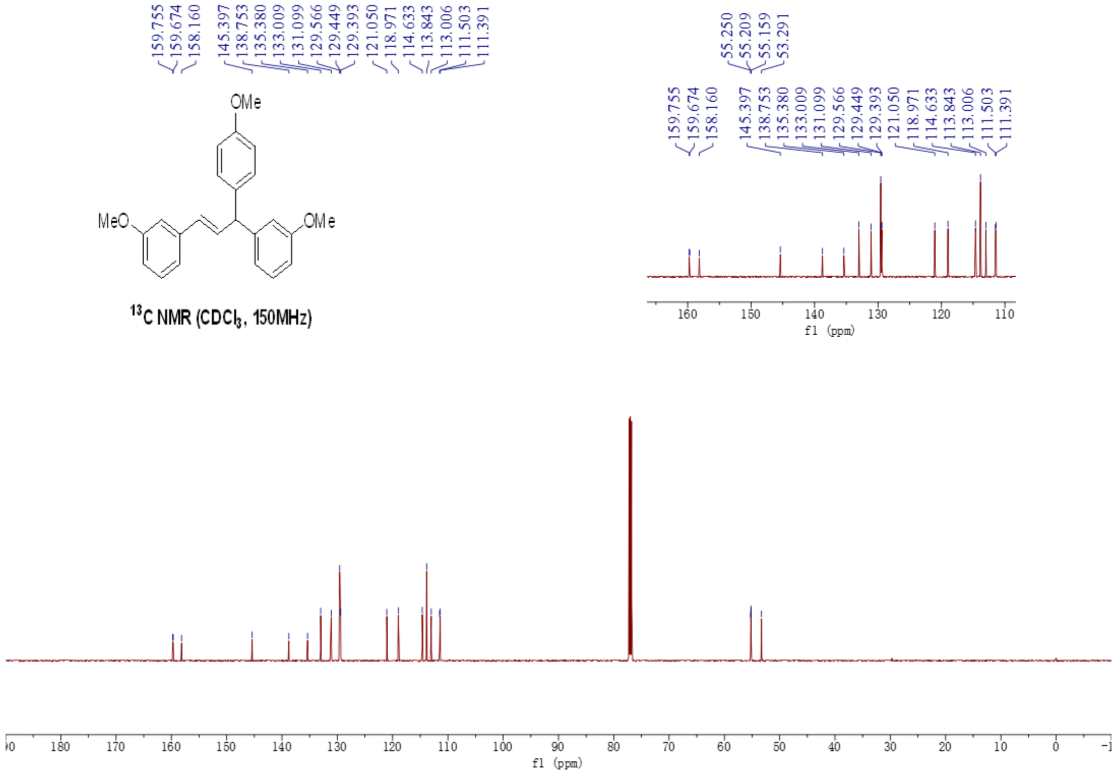

^{13}C NMR ($CDCl_3$, 150MHz)

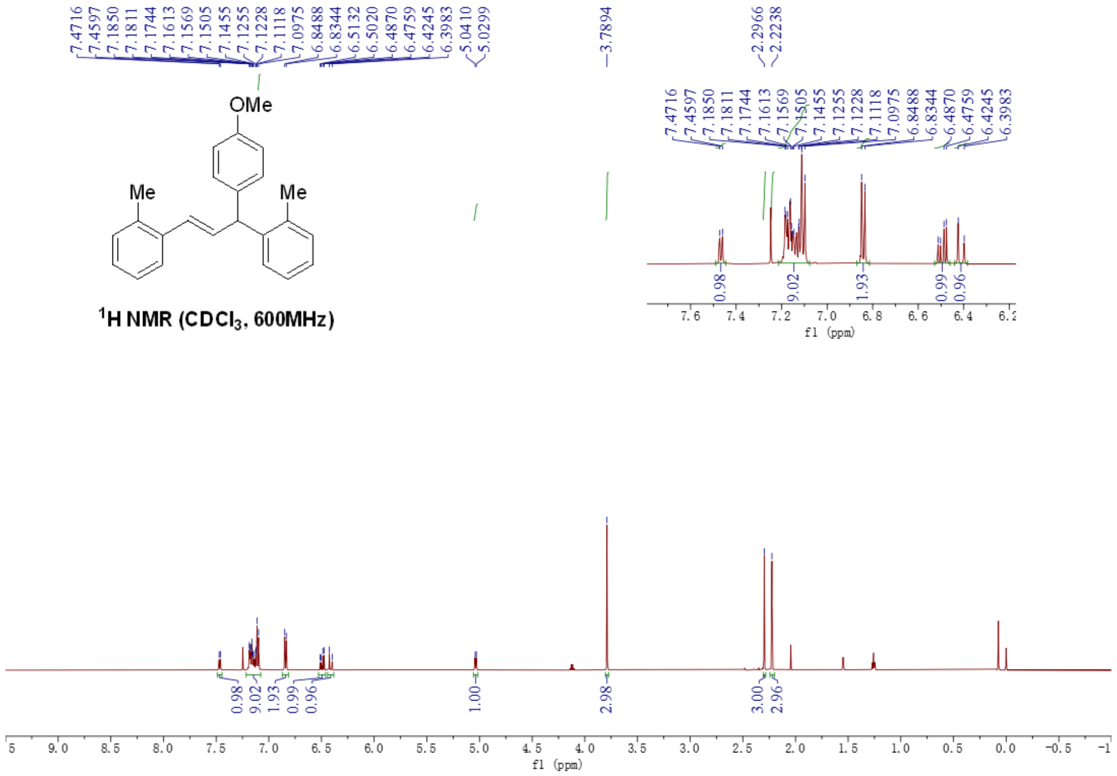

^{1}H NMR ($CDCl_3$, 600MHz)

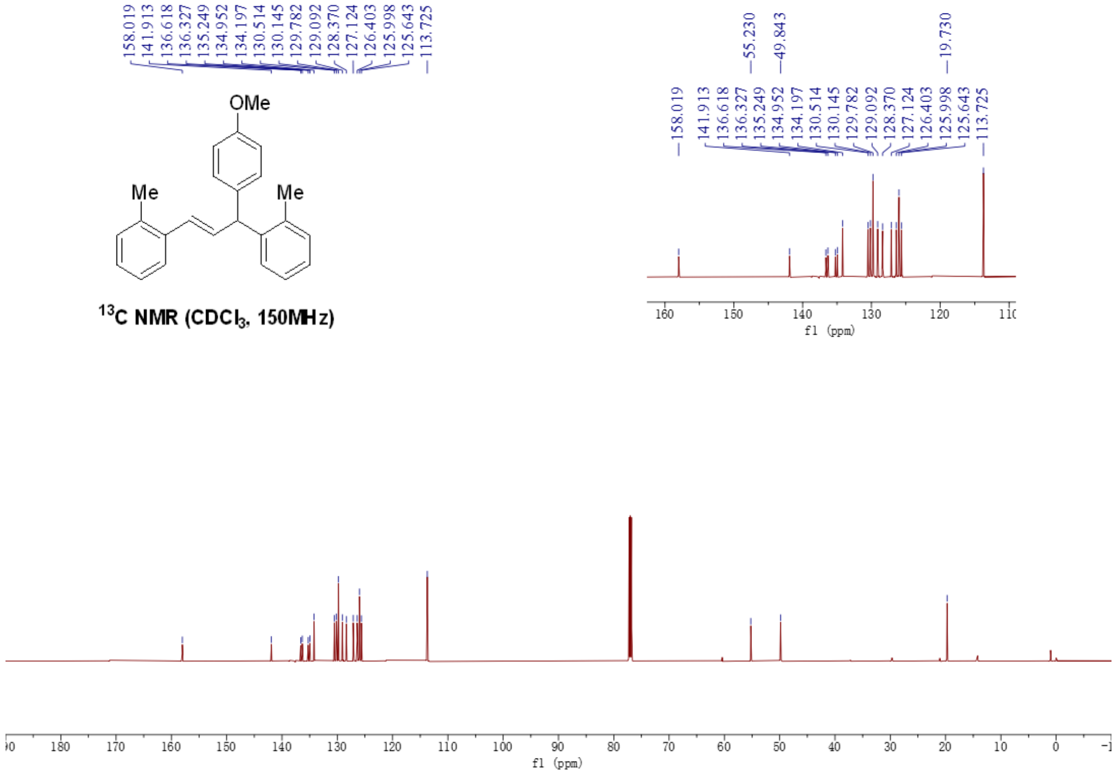

^{13}C NMR ($CDCl_3$, 150MHz)

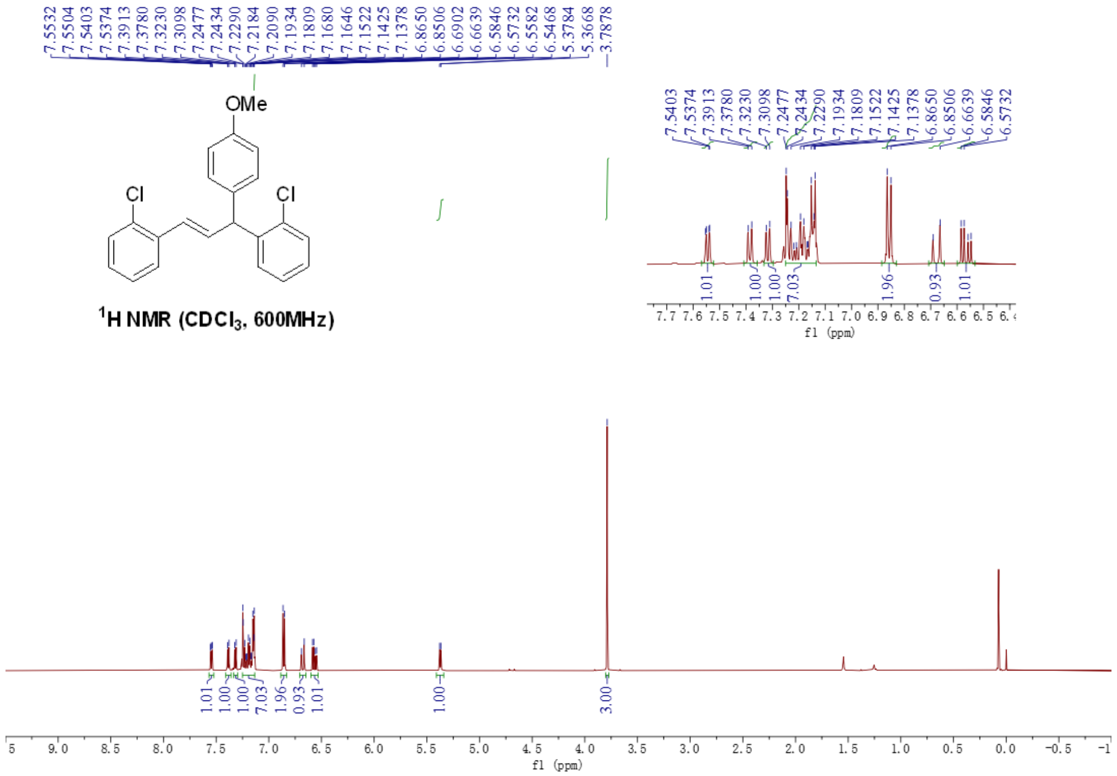

^{1}H NMR ($CDCl_3$, 600MHz)

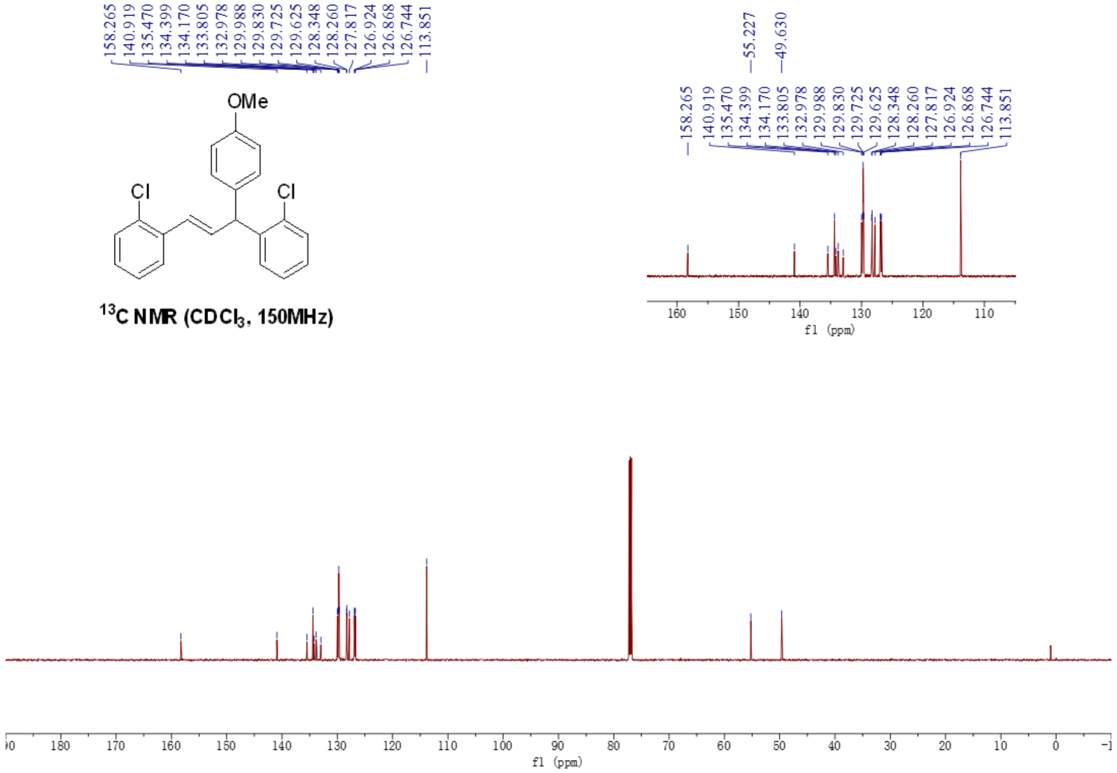

^{13}C NMR ($CDCl_3$, 150MHz)

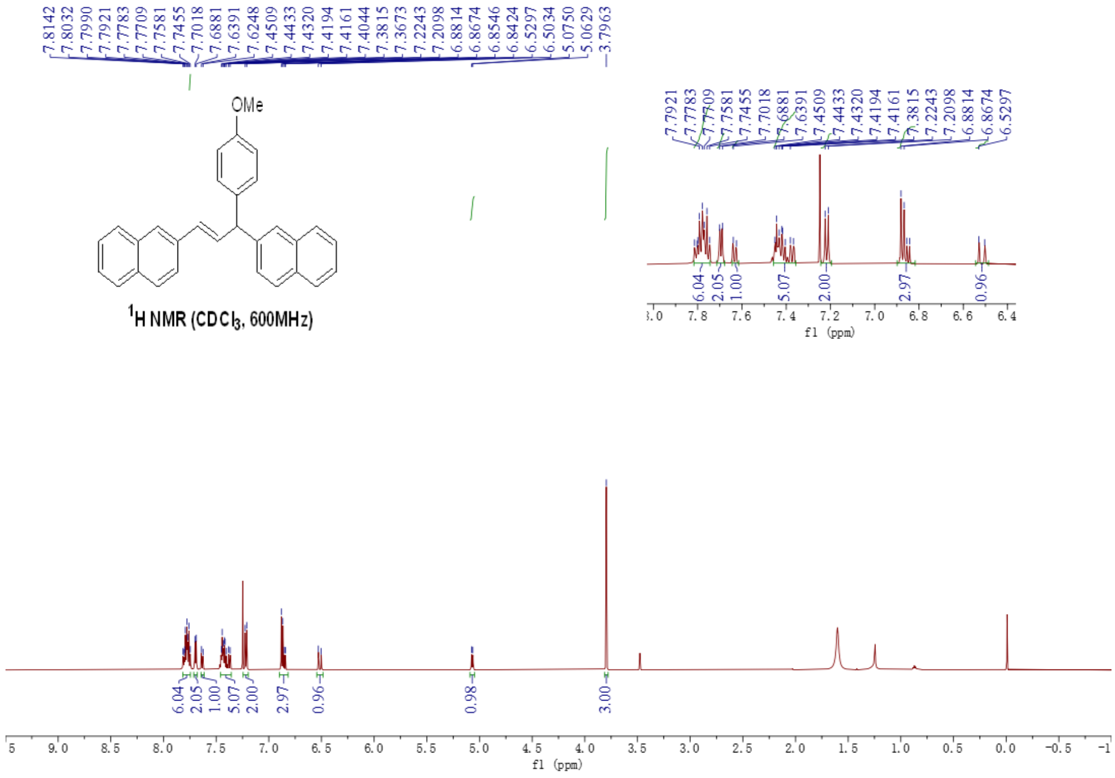

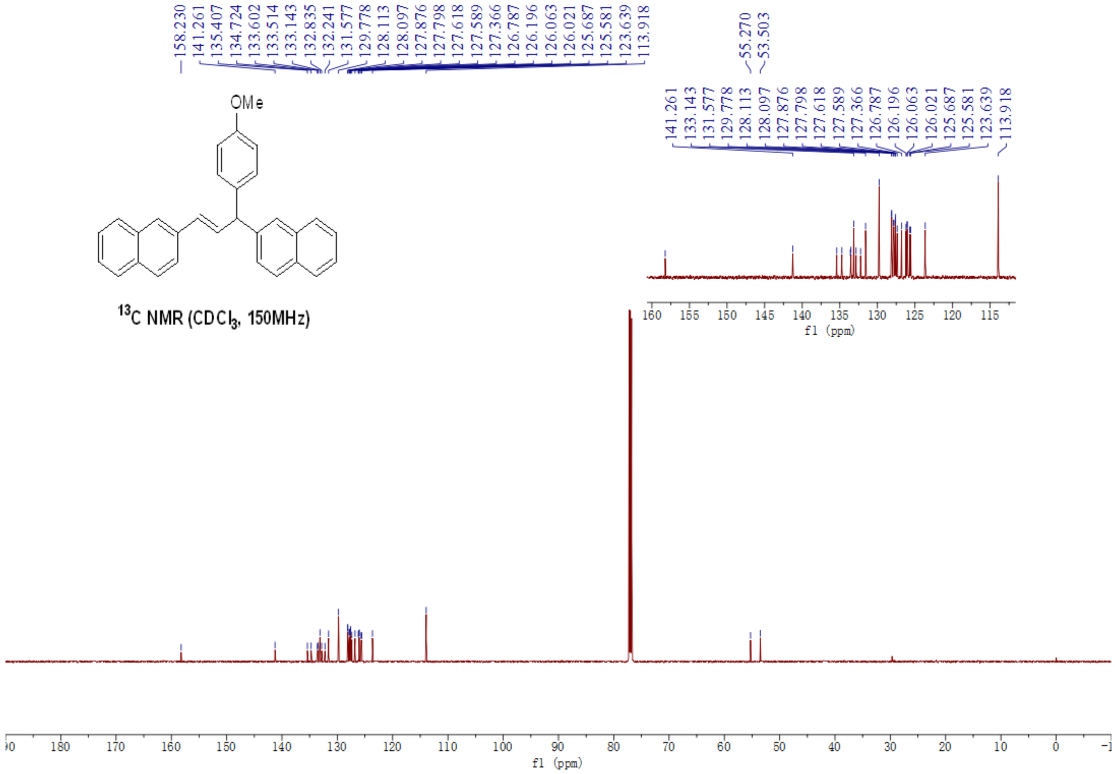

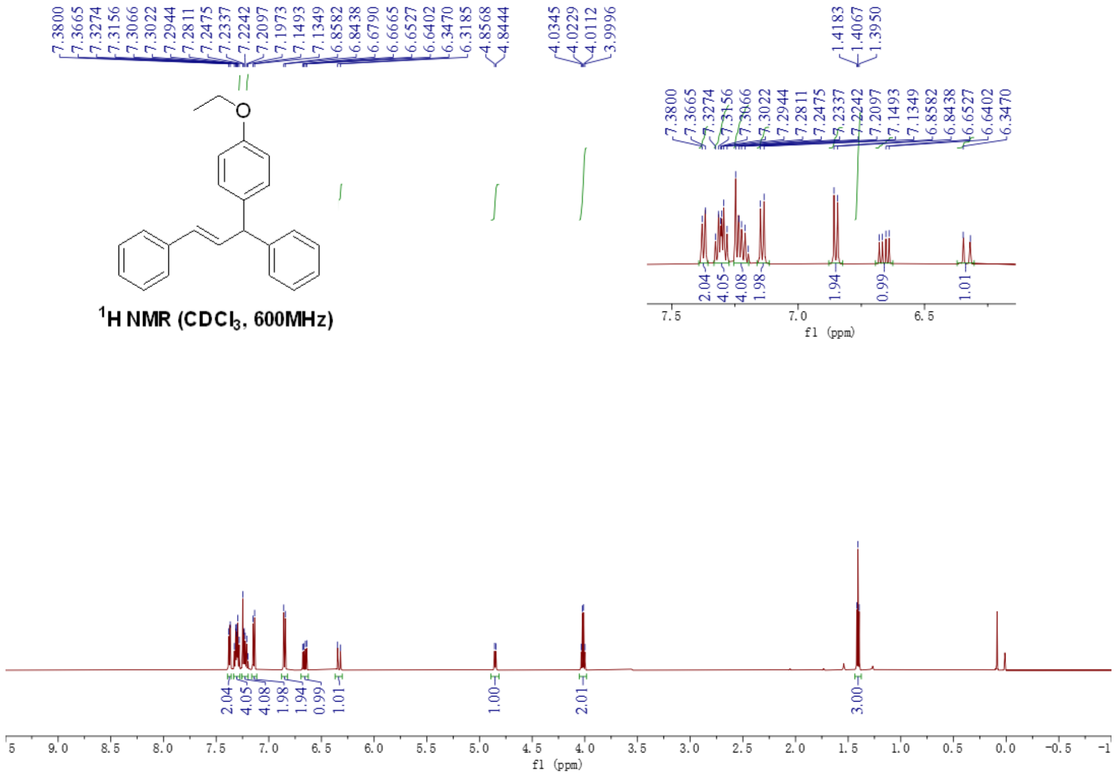

^{1}H NMR ($CDCl_3$, 600MHz)

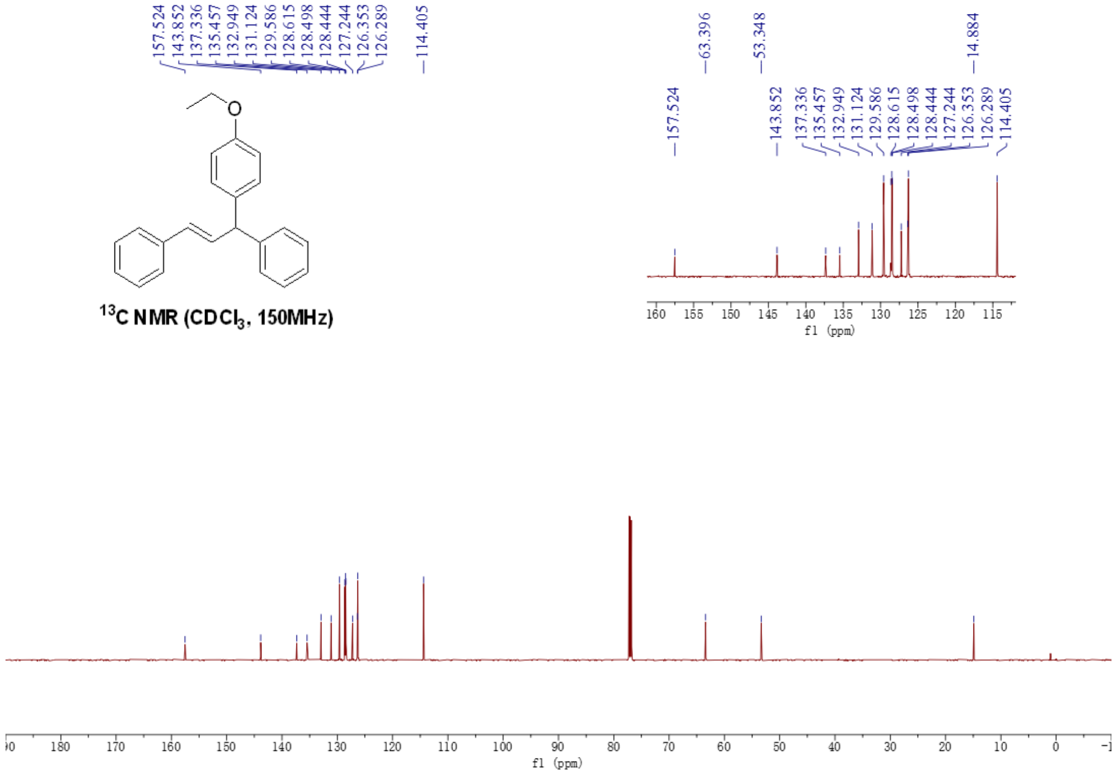

^{13}C NMR ($CDCl_3$, 150MHz)

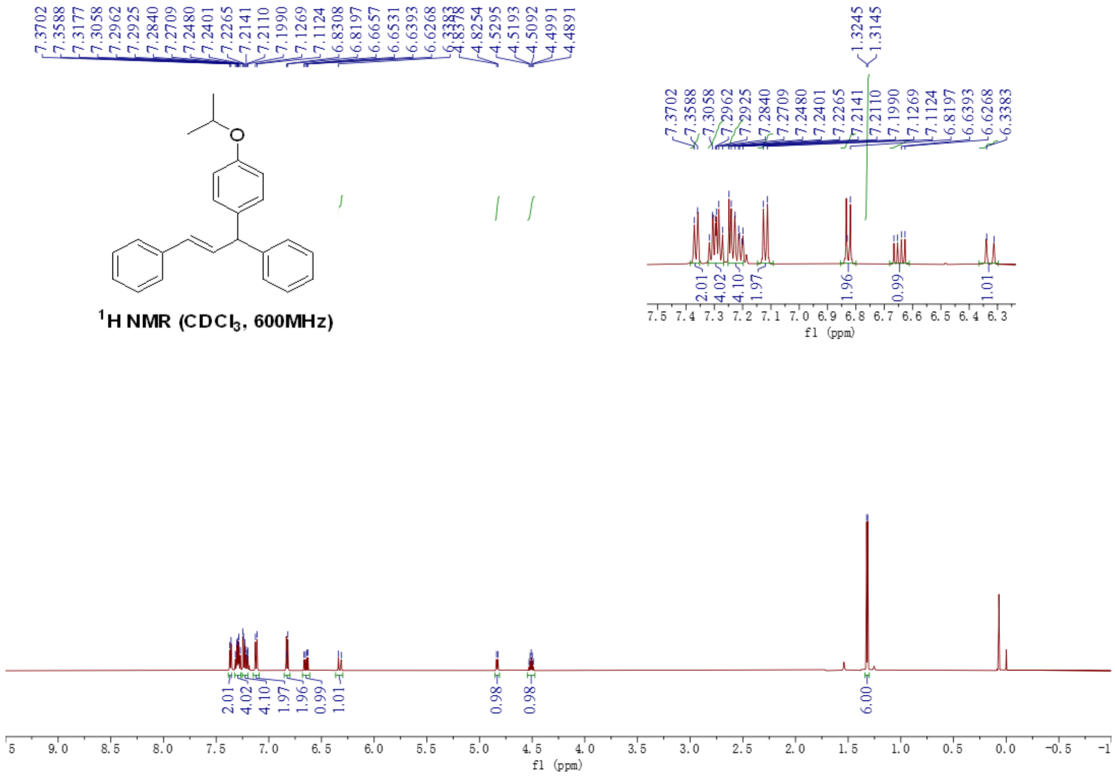

^{1}H NMR ($CDCl_3$, 600MHz)

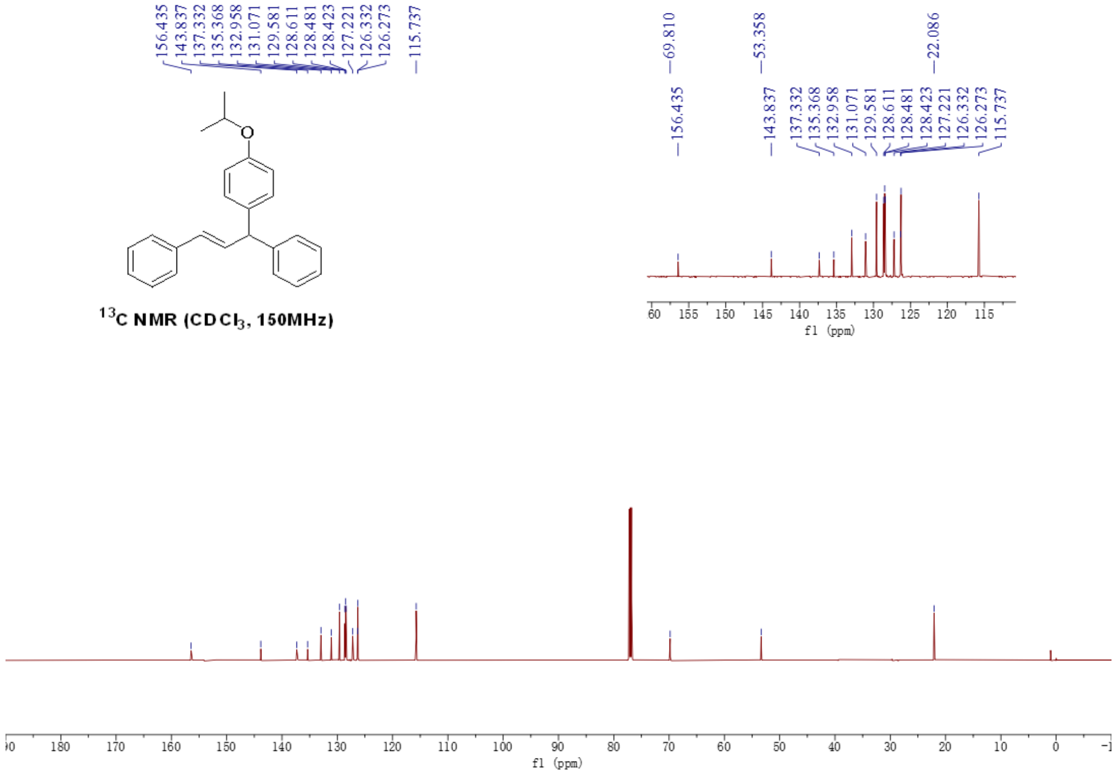

^{13}C NMR ($CDCl_3$, 150MHz)

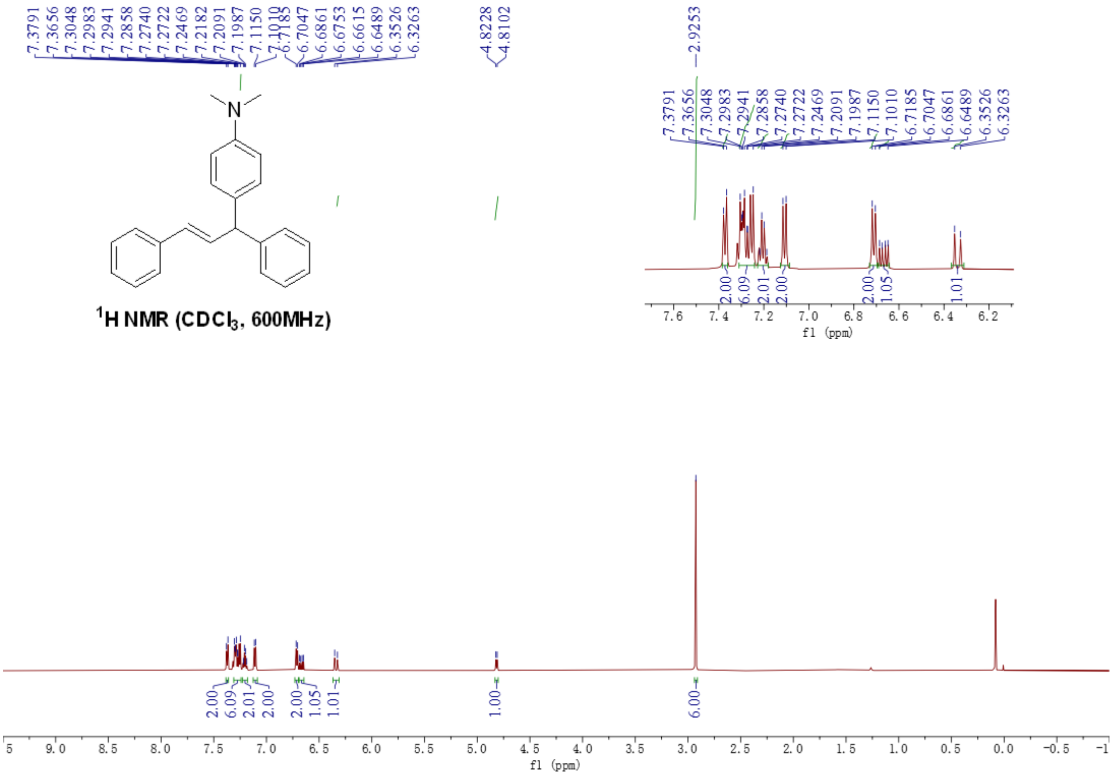

^{1}H NMR ($CDCl_3$, 600MHz)

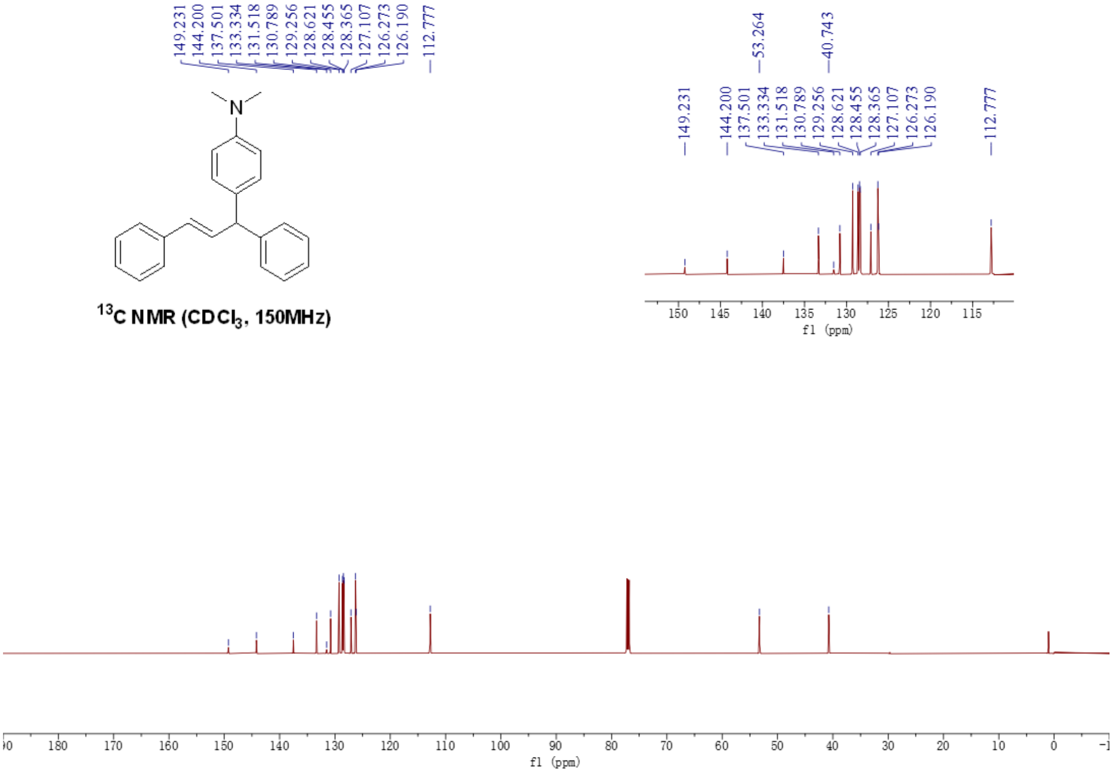

^{13}C NMR ($CDCl_3$, 150MHz)

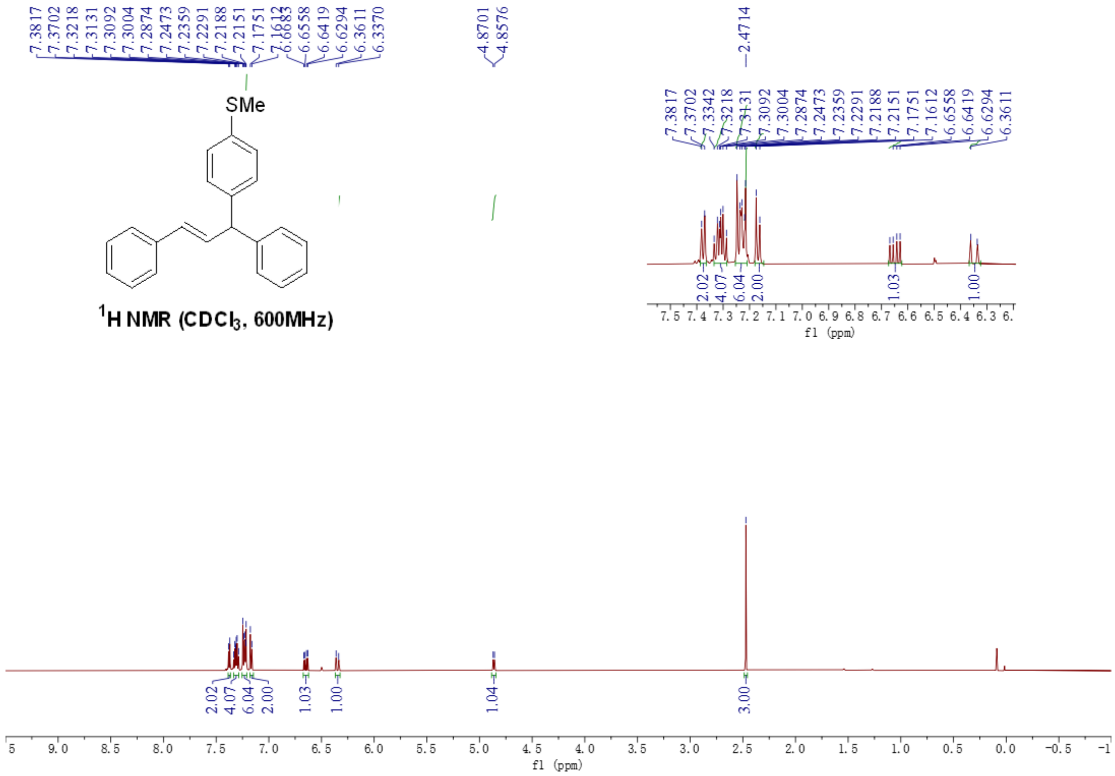

^{1}H NMR (CDCl$_3$, 600MHz)

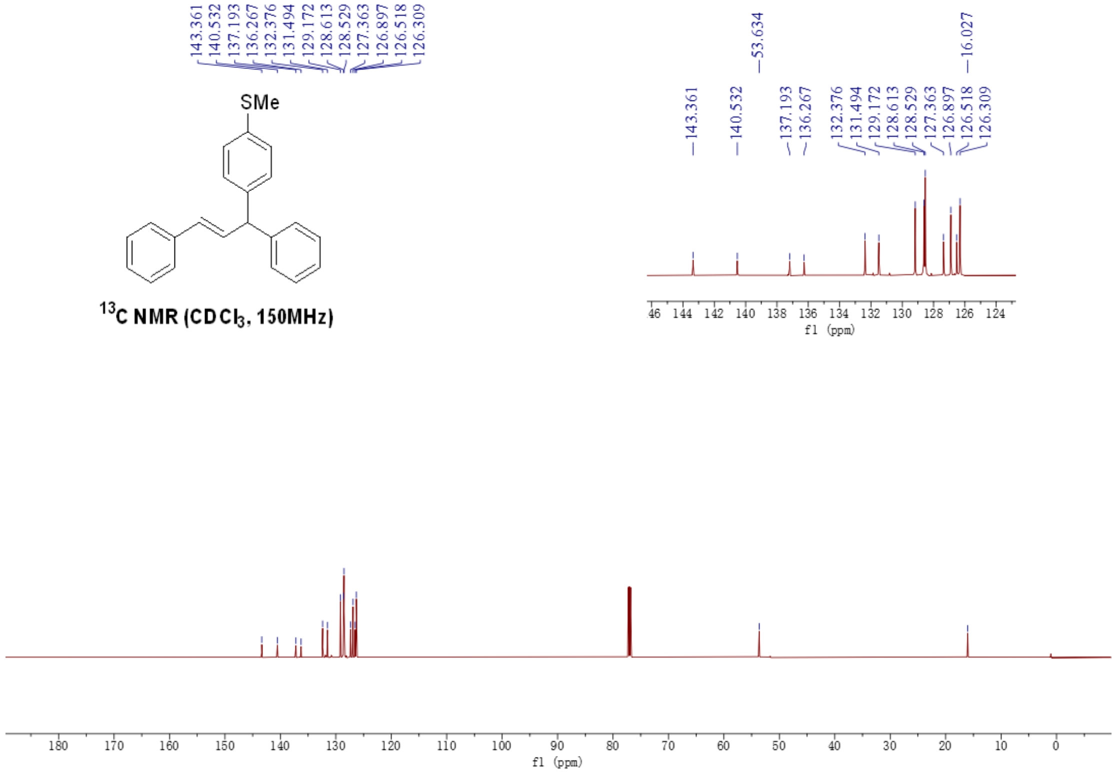

^{13}C NMR (CDCl$_3$, 150MHz)

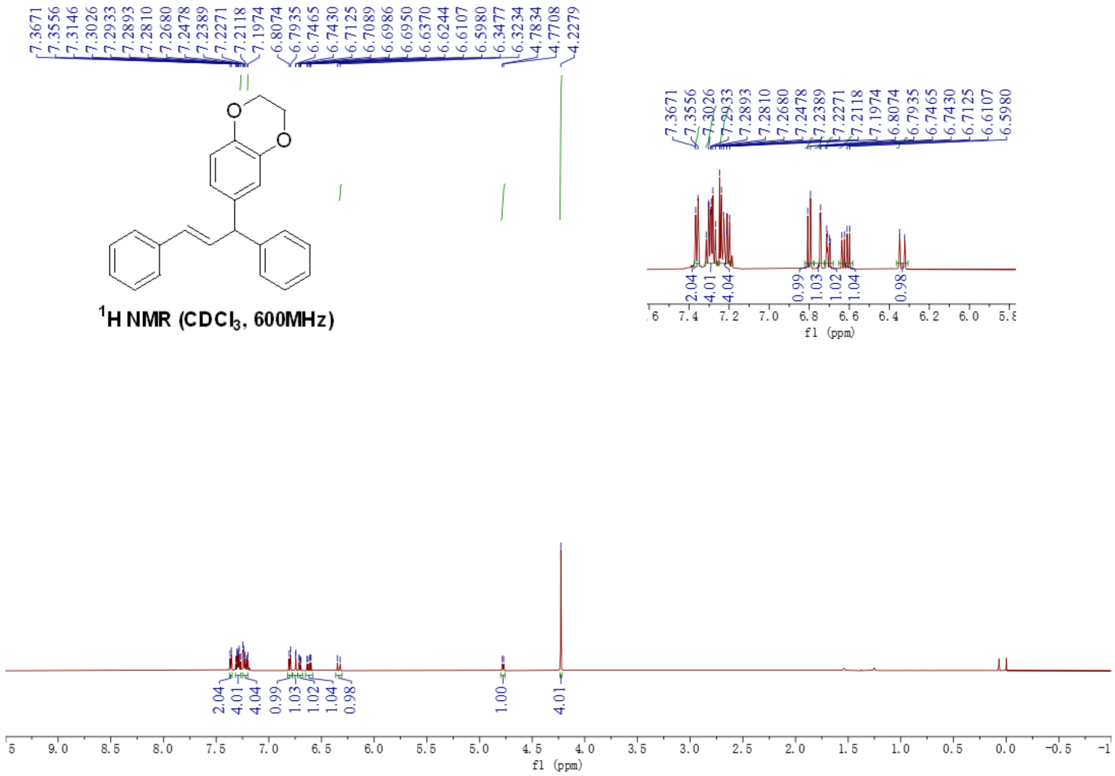
7.3671
7.3556
7.3146
7.3026
7.2933
7.2893
7.2810
7.2680
7.2478
7.2389
7.2271
7.2118
7.1974
6.8074
6.7935
6.7465
6.7430
6.7125
6.7089
6.6986
6.6950
6.6370
6.6244
6.6107
6.5980
6.3477
6.3234
4.7834
4.7708
4.2279
O
O
1H NMR (CDCl3, 600MHz)
2.04
4.01
4.04
0.99
1.03
1.02
1.04
0.98
1.00
4.01
f1 (ppm)

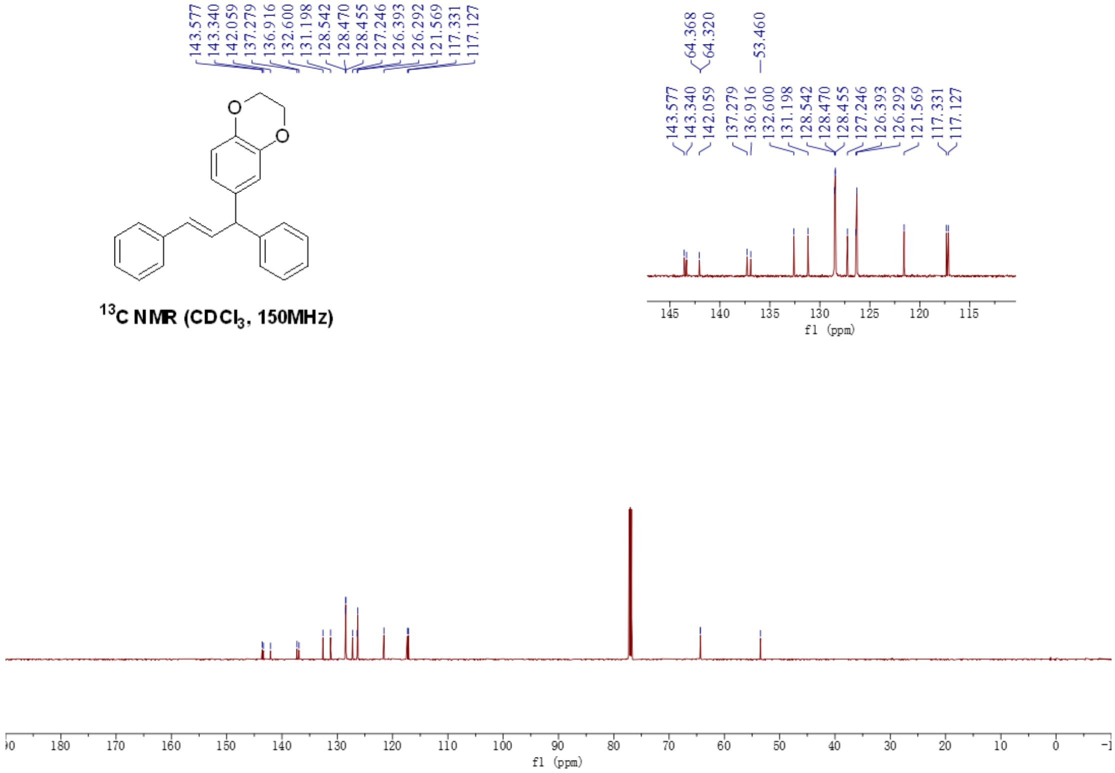
143.577
143.340
142.059
137.279
136.916
132.600
131.198
128.542
128.470
128.455
127.246
126.393
126.292
121.569
117.331
117.127
64.368
64.320
53.460
O
O
13C NMR (CDCl3, 150MHz)
f1 (ppm)

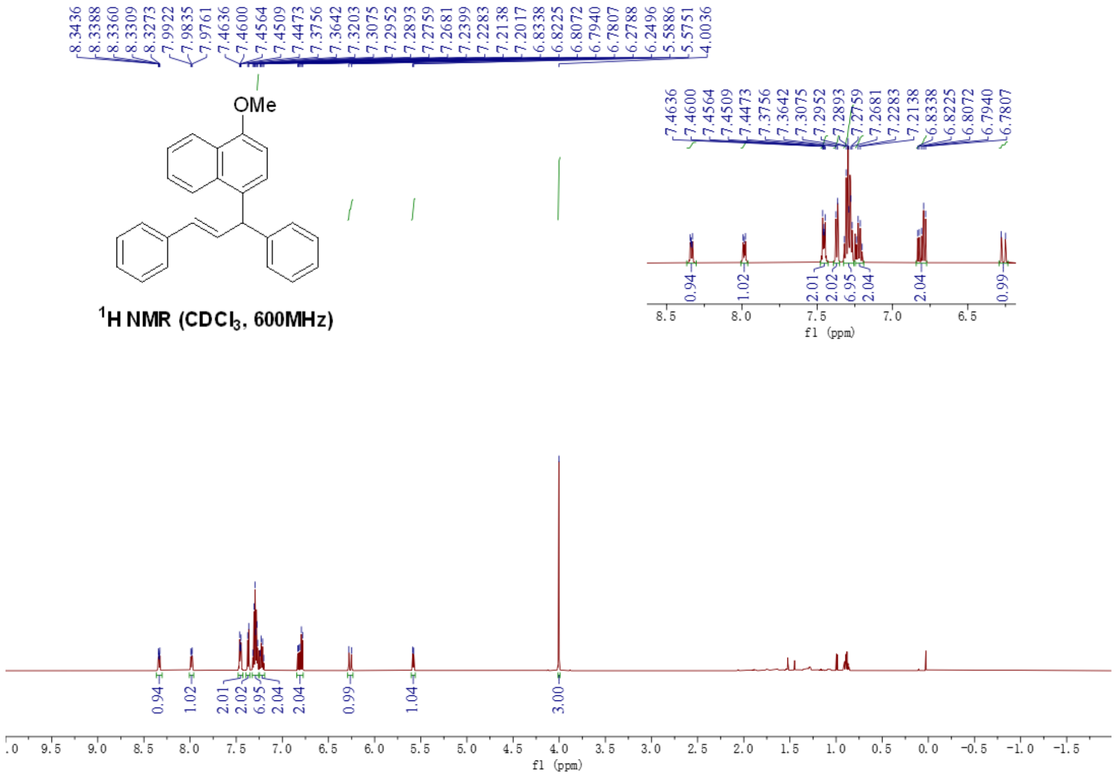

^{1}H NMR ($CDCl_3$, 600MHz)

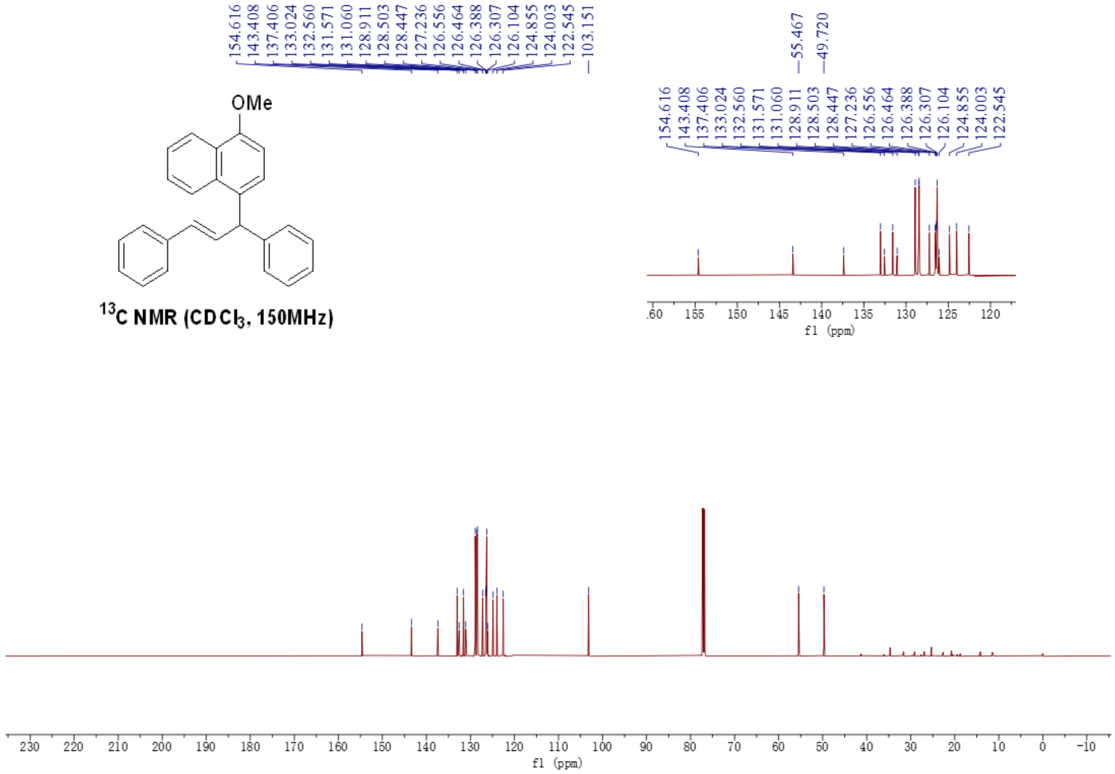

^{13}C NMR ($CDCl_3$, 150MHz)

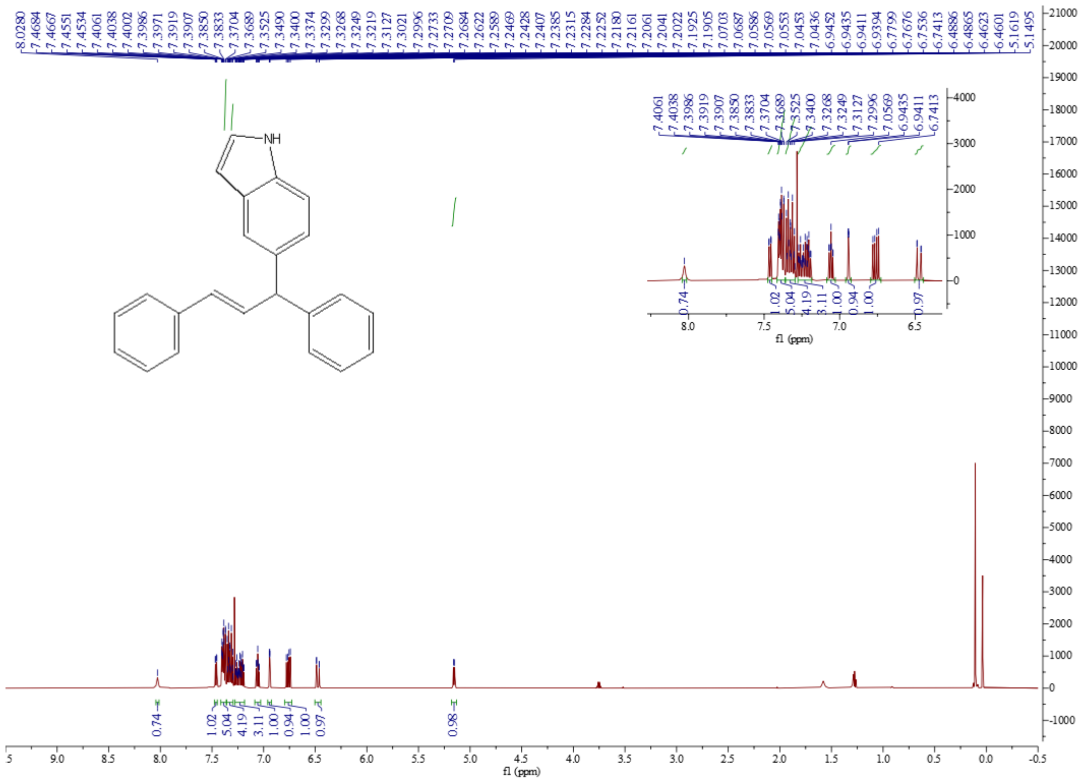

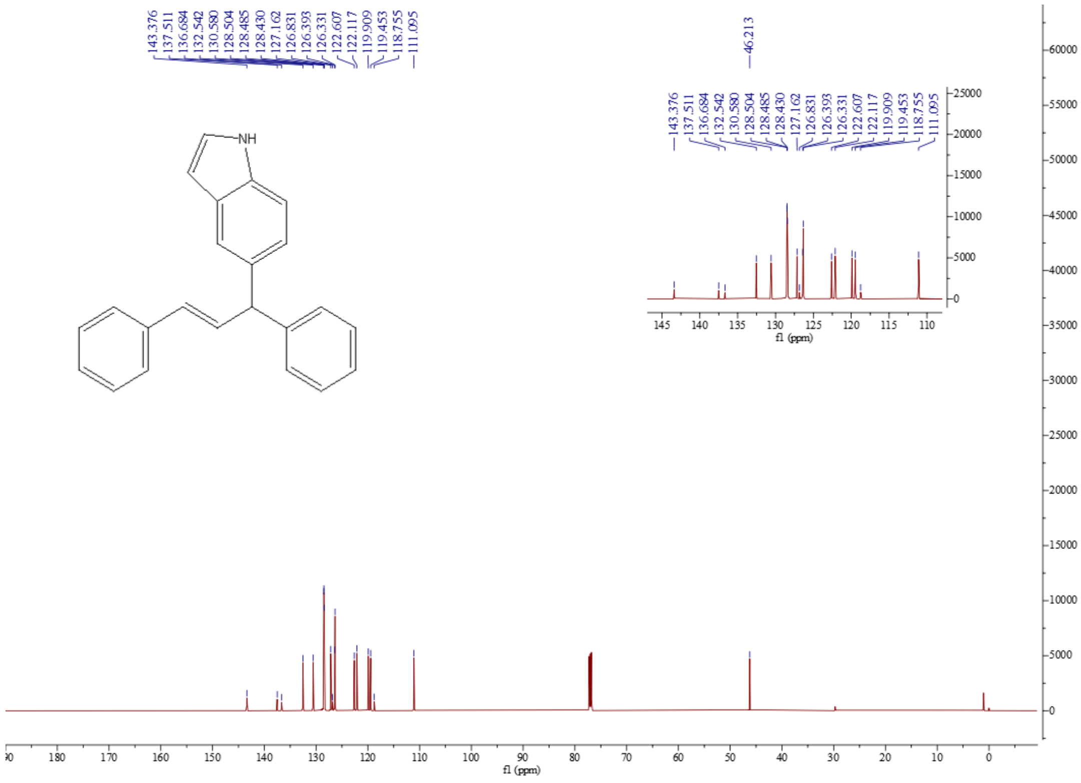

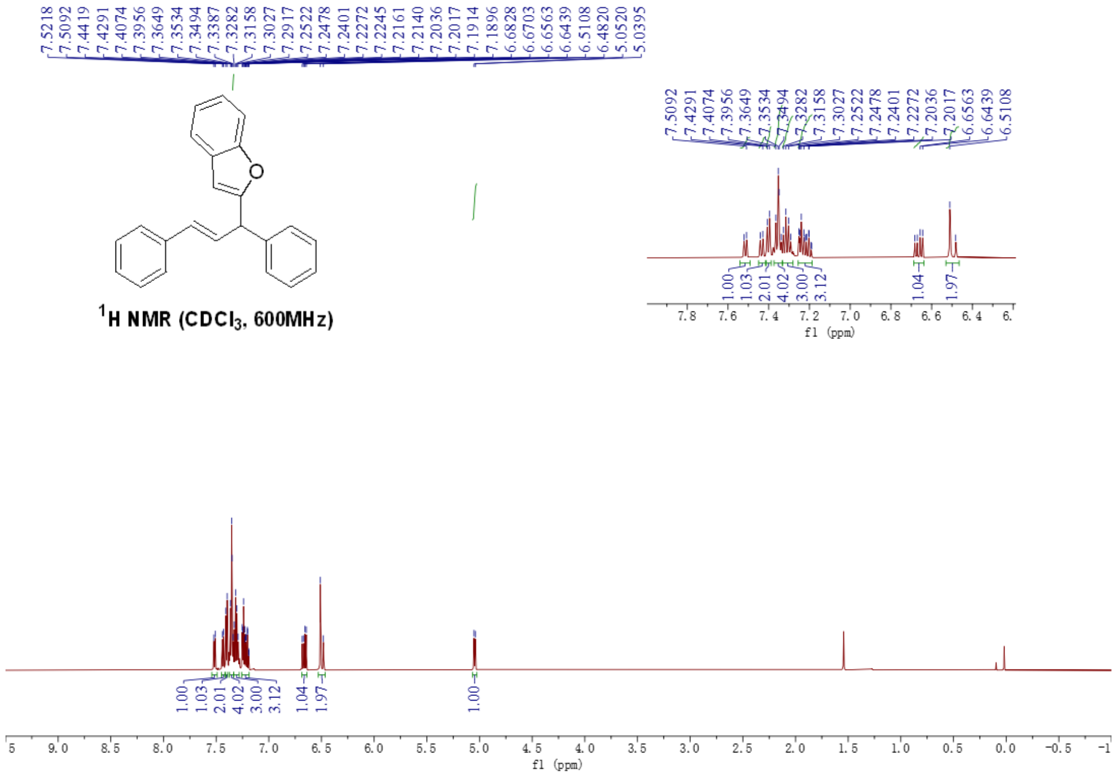

^{1}H NMR ($CDCl_3$, 600MHz)

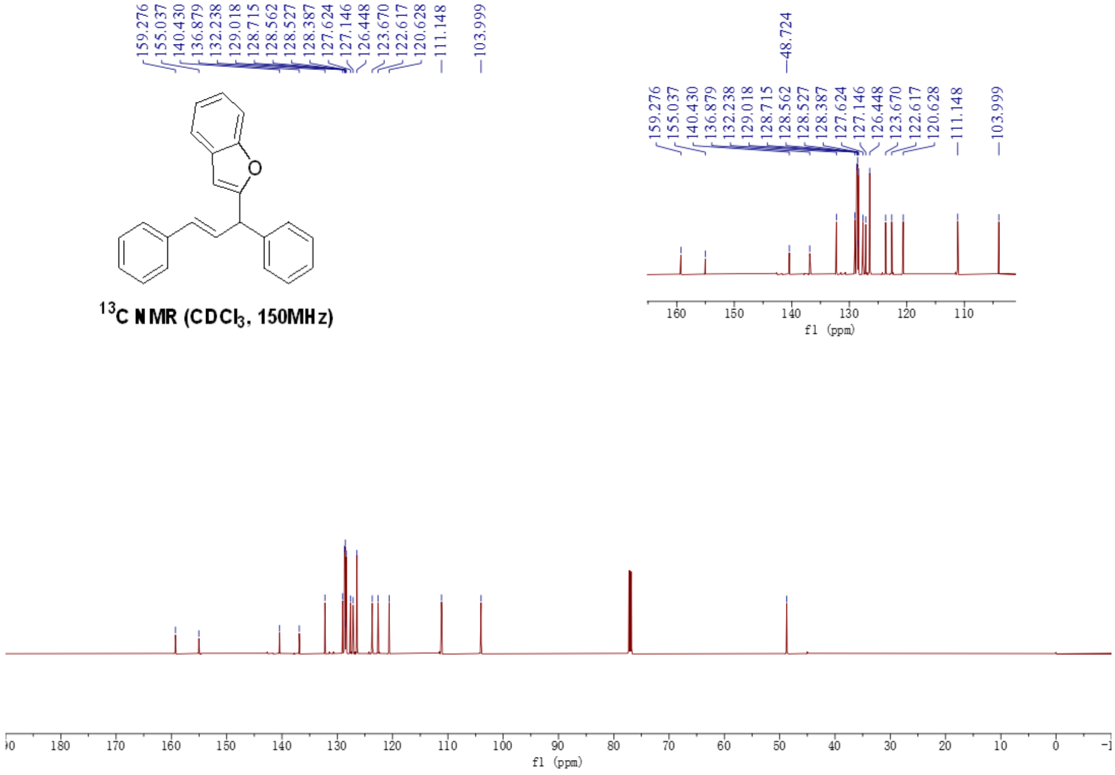

^{13}C NMR ($CDCl_3$, 150MHz)

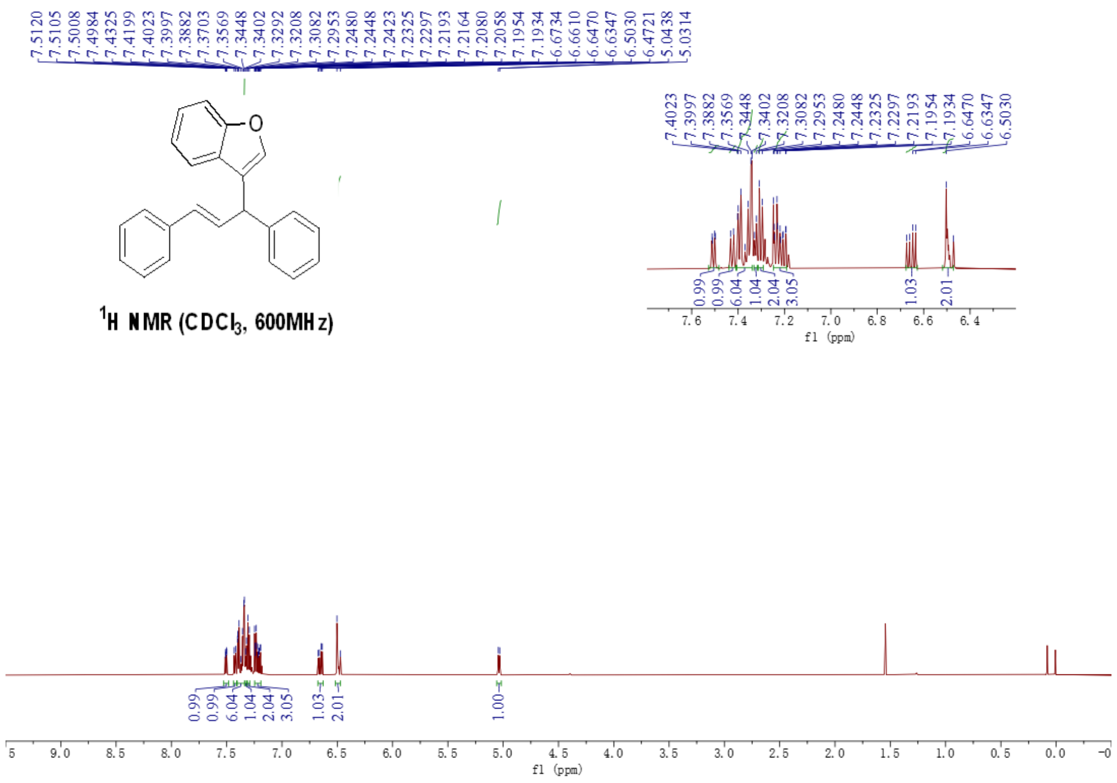

^{1}H NMR ($CDCl_3$, 600MHz)

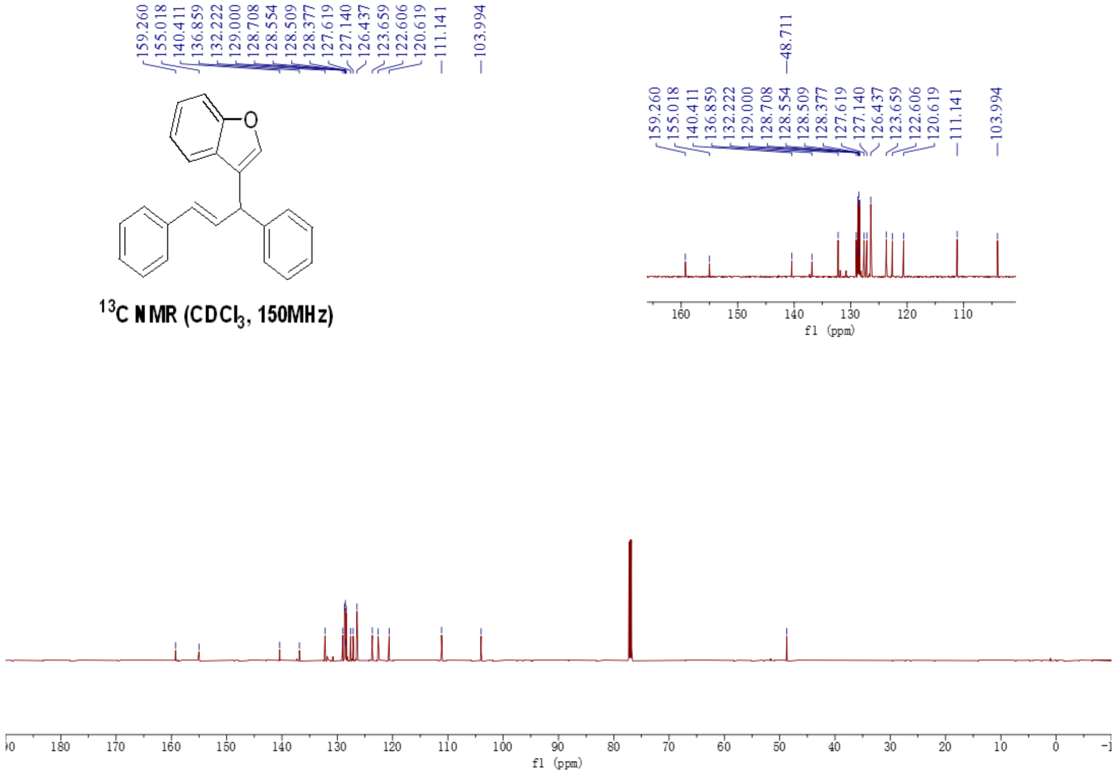

^{13}C NMR ($CDCl_3$, 150MHz)

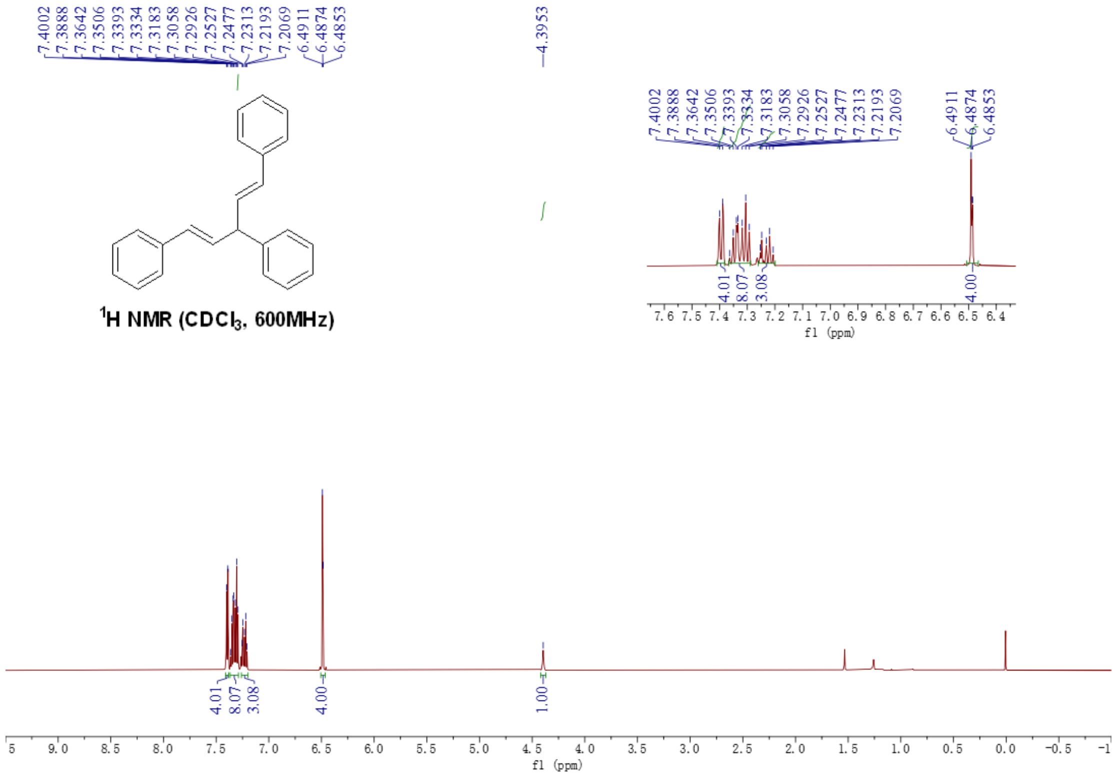
7.4002
7.3888
7.3642
7.3506
7.3393
7.3334
7.3183
7.3058
7.2926
7.2527
7.2477
7.2313
7.2193
7.2069
6.4911
6.4874
6.4853
4.3953
1H NMR (CDCl3, 600MHz)
4.01
8.07
3.08
4.00
1.00
f1 (ppm)

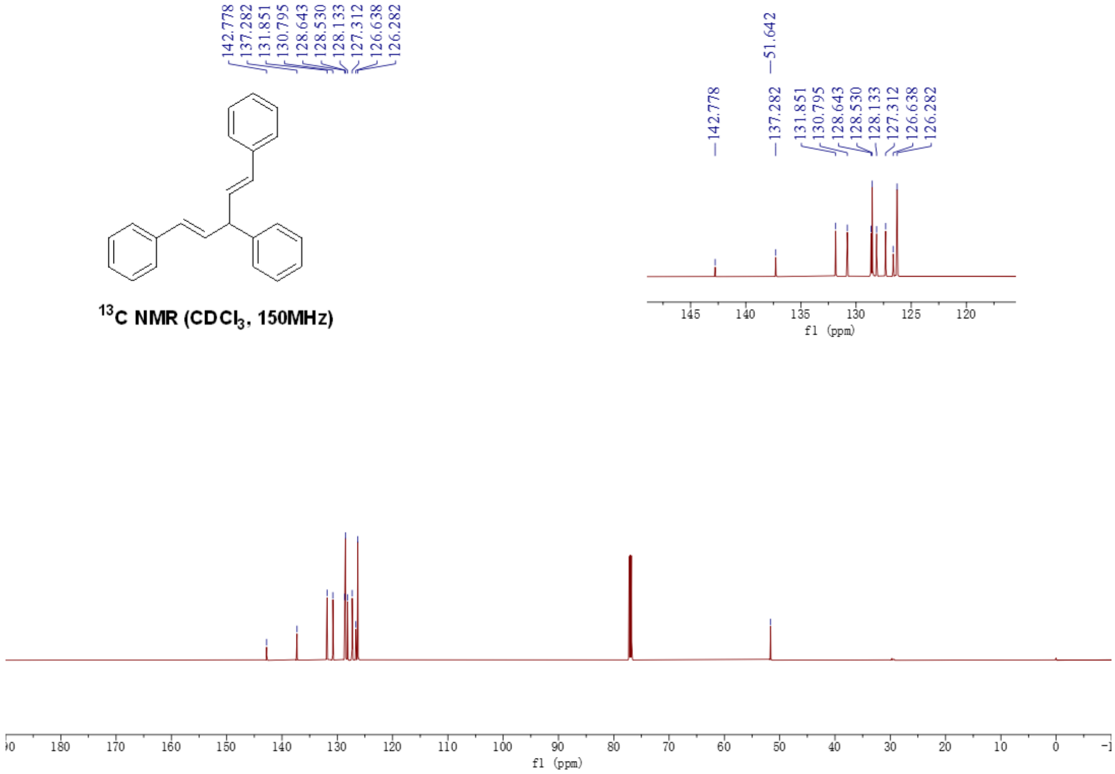
142.778
137.282
131.851
130.795
128.643
128.530
128.133
127.312
126.638
126.282
51.642
13C NMR (CDCl3, 150MHz)
f1 (ppm)
